# Direct OC-CHO coupling towards highly C_2+_ products selective electroreduction over stable Cu^0^/Cu^2+^ interface

**DOI:** 10.1038/s41467-023-43182-6

**Published:** 2023-11-24

**Authors:** Xin Yu Zhang, Zhen Xin Lou, Jiacheng Chen, Yuanwei Liu, Xuefeng Wu, Jia Yue Zhao, Hai Yang Yuan, Minghui Zhu, Sheng Dai, Hai Feng Wang, Chenghua Sun, Peng Fei Liu, Hua Gui Yang

**Affiliations:** 1 Key Laboratory for Ultrafine Materials of Ministry of Education, Shanghai Engineering Research Center of Hierarchical Nanomaterials, School of Materials Science and Engineering, East China University of Science and Technology, 130 Meilong Road, Shanghai, 200237 China; 2 State Key Laboratory of Chemical Engineering, School of Chemical Engineering, East China University of Science and Technology, 130 Meilong Road, Shanghai, 200237 China; 3 Key Laboratory for Advanced Materials and Feringa Nobel Prize Scientist Joint Research Center, Institute of Fine Chemicals, School of Chemistry and Molecular Engineering, East China University of Science and Technology, 130 Meilong Road, Shanghai, 200237 China; 4 Key Laboratory for Advanced Materials, Centre for Computational Chemistry and Research Institute of Industrial Catalysis, School of Chemistry and Molecular Engineering, East China University of Science and Technology, 130 Meilong Road, Shanghai, 200237 China; 5 Department of Chemistry and Biotechnology, and Center for Translational Atomaterials, Swinburne University of Technology, Hawthorn, VIC 3122 Australia

**Keywords:** Electrocatalysis, Electrocatalysis

## Abstract

Electroreduction of CO_2_ to valuable multicarbon (C_2+_) products is a highly attractive way to utilize and divert emitted CO_2_. However, a major fraction of C_2+_ selectivity is confined to less than 90% by the difficulty of coupling C-C bonds efficiently. Herein, we identify the stable Cu^0^/Cu^2+^ interfaces derived from copper phosphate-based (CuPO) electrocatalysts, which can facilitate C_2+_ production with a low-energy pathway of OC-CHO coupling verified by in situ spectra studies and theoretical calculations. The CuPO precatalyst shows a high Faradaic efficiency (FE) of 69.7% towards C_2_H_4_ in an H-cell, and exhibits a significant FE_C2+_ of 90.9% under industrially relevant current density (*j* = −350 mA cm^−2^) in a flow cell configuration. The stable Cu^0^/Cu^2+^ interface breaks new ground for the structural design of electrocatalysts and the construction of synergistic active sites to improve the activity and selectivity of valuable C_2+_ products.

## Abstract (web summary)

The electrosynthesis of multicarbon products from CO_2_ is a highly attractive way to utilize emitted CO_2_. Here, the authors report in situ formed Cu^0^/Cu^2+^ interfaces from copper phosphate-based electrocatalysts for promoting selectivity toward multicarbon products through OC-CHO coupling.

## Introduction

The electrosynthesis of multicarbon (C_2+_) products such as ethanol (C_2_H_5_OH) and ethylene (C_2_H_4_) from CO_2_ is highly attractive because of the versatility of these products in the chemical and energy industries^1–4^. However, the selective production of C_2+_ products from CO_2_ reduction reaction (CO_2_RR) is challenging, with competition from the hydrogen evolution reaction (HER) and C_1_ products (e.g., CO, HCOOH, and CH_4_) production^5–7^. Among all the nanostructured electrocatalysts reported thus far, copper-based materials are known to be the most selective for CO_2_-to-C_2+_ production^8,9^. Up to now, a variety of strategies have been proposed to improve CO_2_-to-C_2+_ selectivity, including controlling oxidation states^10,11^, constructing nanostructures^12,13^, alloying^14,15^, doping^16^, and molecular decorating^1^. Unfortunately, the selectivity of C_2+_ products is still low, especially at high current densities (*j*)^17^.

Recently, manipulating oxidation states for constructing a synergistic Cu^0^/Cu^1+^ interface has been demonstrated as an effective way to promote C_2+_ product conversion via stabilizing Cu^1+^ species^11,12,17–22^. For example, it’s reported that a high concentration of Cu^1+^ (8%) in the iodine-modified Cu catalyst was observed by the linear combination fitting (LCF) analysis of operando X-ray absorption spectra (XAS), which contributed to the high FE_C2+_ of about 80% at the *j* lower than 40 mA cm^−2^ (ref. ^12^). Our group also constructed the stable Cu^0^/Cu^1+^ interface via isolating Cu-S motif with C_2+_ selectivity over 80%^22^. By combining Cu^0^ and Cu^1+^ synergistic species, the adsorption of *CO can take place at different active sites, resulting in the improvement of CO dimerization, i.e., the Cu^0^ site can activate CO_2_ and facilitate the following electron transfers, while the Cu^1+^ site strengthens the *CO adsorption and boosts C-C coupling, further endowing a lower Gibbs free energy for *OCCOH formation^11,21,23–25^. Similarly, it was revealed that over the two adjacent Cu^0^ and positively charged Cu^δ+^ atoms, the Cu^0^ site can adsorb CO_2_ and the neighboring Cu^δ+^ site is conducive to H_2_O adsorption, thus promoting the activation of CO_2_^26^. Moreover, theoretical studies have shown that the subsurface O stabilized surface Cu^δ+^ species indeed enhances the C_2_ products selectivity through increasing the *CO coverage^27^.

In addition to the widely reported Cu^1+^ species in the field of CO_2_RR, Cu^2+^ species with higher oxidation states than Cu^0^ or Cu^1+^ sites feature the structure characteristics to easily bind CO or H_2_O, which have been demonstrated in other heterogenous catalysis fields^28–30^. Recently, Qiao et al. have proved the feasibility of Cu^2+^ sites for promoting *CO hydrogenation in the Cu-Ce-O_x_ solid solutions, which facilitate the generation of *CHO intermediates instead of *CO dimerization on the single Cu^2+^ sites^31^. As a consequence, Cu-Ce-O_x_ delivered a high selectivity for CH_4_ with significant suppression of the C_2_ products. The above findings suggest the possibility of retaining Cu^2+^ sites through material structure design and promoting *CHO intermediate formation on Cu^2+^ catalytic active sites. Inspired by these works, we anticipate that the construction of Cu^0^/Cu^2+^ interface may provide the possibility of direct OC–CHO coupling process compared with single Cu^2+^ sites; however, it still remains a grand challenge to build stable Cu^0^/Cu^2+^ interface under operando CO_2_RR conditions.

Herein, we rationally screened the Cu-based precatalysts for constructing stable Cu^0^/Cu^2+^ interfaces, and elucidated their synergic roles in CO_2_-to-C_2+_ conversion. Using Materials Project Database (MPD) and density functional theoretical (DFT) calculations therewith, we screened different Cu^2+^-containing compounds and found Cu^2+^ phosphorus oxysalts of Cu_2_P_2_O_7_ and Cu_3_(PO_4_)_2_ (collectively named CuPO) to be the most promising candidate for exhibiting the highest stability of Cu^0^/Cu^2+^ interface under the electroreduction condition; in consequence, the peculiar Cu^0^/Cu^2+^ interfaces facilitate a low-energy pathway of *CHO coupling with *CO to form *OCCHO intermediate. Experimentally, we synthesized CuPO catalysts through a facile and scalable method, which achieved 69.7% FE for C_2_H_4_ in the neutral electrolyte, and 90.9% FE_C2+_ with a C_2+_ partial current density (*j*_C2+_) of over 300 mA cm^−2^ in a flow cell using the alkaline electrolyte. Our operando experimental characterizations unambiguously demonstrate the robust existence of Cu^0^/Cu^2+^ interfaces derived from CuPO during CO_2_RR, and in situ surface-enhanced infrared absorption spectroscopy (SEIRAS) in conjunction with DFT calculation testify that the Cu^2+^ sites are conducive to the formation of *CHO, which then facilely coupled with *CO on Cu^0^ surface to form *OCCHO intermediate, leading to high-efficiency CO_2_-to-C_2+_ conversion performance.

## Results

### Theoretical calculation

**Fig. 1:**
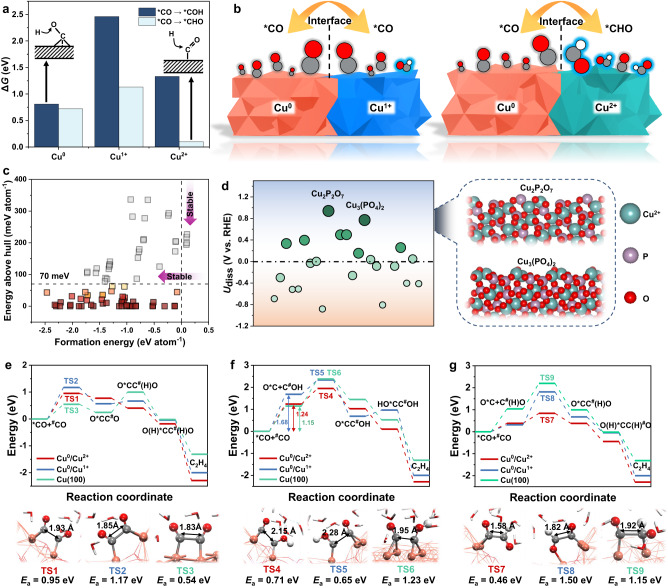
Theoretical calculations. **a** Reaction energy of *CO hydrogenation to *COH or *CHO on Cu, Cu^1+^ and Cu^2+^ sites, respectively. **b** Schematic diagram of different intermediates (CO or CHO) on the Cu^0^/Cu^1+^ and Cu^0^/Cu^2+^ interfaces. **c** Thermodynamic stabilities of materials as a function of *E*_f_ and *E*_hull_. **d** Electrochemical stability of candidates, where *U*_diss_ is the dissolution potential of materials, larger and greener circles represent more stable materials (i.e., larger *U*_diss_), and the structures of the most stable Cu_2_P_2_O_7_ and Cu_3_(PO_4_)_2_. **e**–**g** Energy profiles of different C–C coupling processes (* and ^#^ represent Cu^0^ and Cu^2(1)+^ sites, respectively), **e** CO-CO, **f** COH-CO, and **g** CHO-CO at the Cu^0^/Cu^2(1)+^ interfaces and Cu(100), and the related transition state structures (TS1 ~ TS9) of different C-C coupling processes.

The formations of *CO, *COH, and *CHO intermediates on Cu^0^, Cu^1+^ and Cu^2+^ sites induce different coupling manners and probabilities for C_2+_ species formation on Cu^0^/Cu^1+^ and Cu^0^/Cu^2+^, which provide guidance on designing the Cu-based catalysts for CO_2_RR to C_2+_ products^11,31^. Firstly, we explored the formation energy of *CHO or *COH (*CO + H^+^/e → *CHO or *COH) on classical Cu^0^ (metallic Cu), Cu^1+^ (Cu_2_O), and Cu^2+^ (CuO) sites, respectively, which play key roles in the C_2+_ products. From Fig. 1a, one can see that on the Cu^0^ and Cu^1+^ sites, the *CHO or *COH formation is relatively endothermic at the potential *U* = 0 V (all potentials were calibrated to the reversible hydrogen electrode (RHE) if not mentioned), indicating that *CO could be the primary intermediates. By comparison, the Cu^2+^ site has an excellent ability to hydrogenate *CO to form *CHO (Δ*G* = 0.10 eV) rather than *COH (Δ*G* = 1.33 eV). Accordingly, it can be expected that the Cu^0^ and Cu^1+^ sites could be covered by CO intermediates, while the Cu^2+^ site contributes to *CO hydrogenation to form *CHO intermediates that can facilitate the direct OC–CHO coupling process to increase the selectivity of C_2+_ product (Fig. 1b).

Facing various Cu-based catalysts with Cu^2+^ sites, it is difficult and time-consuming to examine each one and locate the optimal one with superior electrochemical stability and catalytic performance using complex experimental methods. High-throughput screening as an efficient forecast method for a large group of candidate materials has been widely used to predict promising materials for experimental synthesis. Here, we extracted 83 Cu^2+^-containing compounds from Materials Project, and various criteria were utilized to filtrate candidates (details in the Supplementary Note 1). Firstly, the thermodynamic stability that is the intrinsic property of materials was considered, in which two criteria were used: (i) the formation energy (*E*_f_) should be less than 0 eV, and the more negative *E*_f_ means the higher thermodynamic stability; (ii) the energy above the convex hull (*E*_hull_) should be less than 70 mV/atom^32^, which can further quantify the structural stability of materials, and the smaller *E*_hull_ indicates that the corresponding material is more stable. Accordingly, 26 candidates were screened out (Fig. 1c). Then, their electrochemical dissolution potential *U*_diss_ was further assessed (see details in the Supplementary Note 2), and a more positive *U*_diss_ means higher electrochemical stability. As shown in Fig. 1d, it can be found that 9 Cu^2+^-containing compounds exhibit good electrochemical stability, in which Cu_2_P_2_O_7_ (*U*_diss_ = 0.94 V) and Cu_3_(PO_4_)_2_ (*U*_diss_ = 0.77 V) process the optimal electrochemical stability. Meanwhile, the high formation energy of the O_vac_ on Cu_3_(PO_4_)_2_ (0.82 eV) indicated that the phosphate group could play an important role in stabilizing the O atoms, thus limiting the reduction of Cu^2+^ (Supplementary Fig. 3). Therefore, Cu_2_P_2_O_7_ and Cu_3_(PO_4_)_2_ could be two promising candidates to construct Cu^0^/Cu^2+^ interface for CO_2_RR to C_2+_ products under the suitable reaction conditions.

In order to clarify the performance of the Cu^0^/Cu^2+^ interface in catalyzing CO_2_RR to produce C_2+_ products, we selected Cu_2_P_2_O_7_ and Cu_3_(PO_4_)_2_ as examples to construct the Cu^0^/Cu^2+^ interface (Supplementary Fig. 1), and explored its ability to promote the C–C bond coupling in comparison with the common Cu^0^/Cu^1+^ interface and the classical Cu(100) surface (Fig. 1e, f). Here, we considered all three general collaborative pathways: (i) *CO + ^#^CO → OC–CO, (ii) *CO + ^#^COH → OC–COH and (iii) *CO + ^#^CHO → OC–CHO (* and ^#^ represent Cu^0^ and Cu^2(1)+^ sites, respectively)^8^, and calculated their reaction energy energies, aiming to understand the C–C bond coupling mechanism in depth. As shown in Fig. 1e–g, CO_2_RR on the Cu^0^/Cu^2+^ interface always has a lower energy profile than that on the Cu^0^/Cu^1+^ one, regardless of the C-C bond coupling pathways, implying the better acceleration of the Cu^0^/Cu^2+^ interface for the C-C coupling. In addition, although the OC–COH coupling process has a relatively low barrier of 0.71 eV at the Cu^0^/Cu^2+^ interfaces compared with the OC-CO coupling process (*E*_a_ = 0.95 eV), COH formation is difficult and requires amount of energy, leading the high effective energy barrier (Fig. 1f). The formation of CHO is much easier compared with that of COH on Cu^2+^ sites; importantly, the ^#^CHO intermediate only needs to overcome a low-energy barrier of only 0.46 eV to couple with *CO at the Cu^0^/Cu^2+^ interface (Fig. 1g), which is also lower than the energy barrier (0.54 eV) for OCCO dimerization on classical Cu(100). These indicate that the OC–CHO coupling process at the Cu^0^/Cu^2+^ interface constructed by Cu_2_P_2_O_7_ (or Cu_3_(PO_4_)_2_, Supplementary Fig. 2) has an evident superiority over the OC–CO or OC–COH coupling processes, and at the same time, the Cu^2+^ site can well facilitate the *CO hydrogenation to the CHO intermediate for high C_2+_ yield instead of C_1_ (Supplementary Fig. 4).

### Catalysts synthesis and structure investigation

**Fig. 2:**
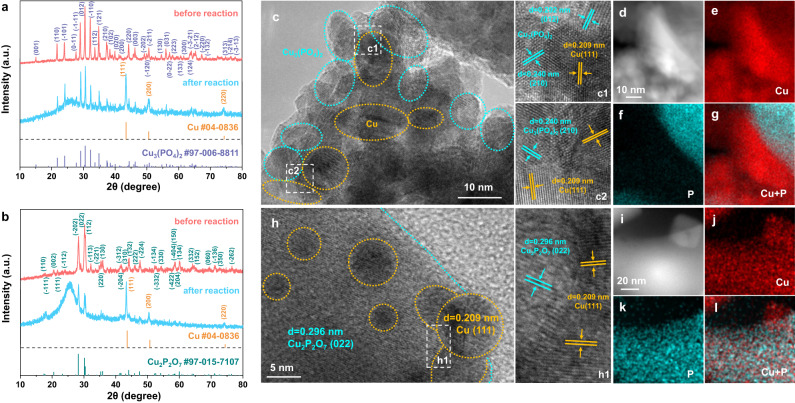
Structural and compositional analyses. **a**, **b** XRD patterns of **a** Cu_3_(PO_4_)_2_ and **b** Cu_2_P_2_O_7_ before and after CO_2_RR at the potential of −1.40 V, indicating the coexistence of CuPO and metallic Cu components in both Cu_3_(PO_4_)_2_ and Cu_2_P_2_O_7_ samples during CO_2_RR. **c** HRTEM images of the Cu_3_(PO_4_)_2_ sample after CO_2_RR at the potential of −1.40 V. **d** Enlarged HAADF-STEM image of Cu_3_(PO_4_)_2_ after reaction and **e**–**g** its corresponding EDS elemental mapping images of **e** Cu, **f** P and **g** mixed elements, respectively. **h** HRTEM images of the Cu_2_P_2_O_7_ sample after CO_2_RR at the potential of −1.40 V. **i** Enlarged HAADF-STEM image of Cu_2_P_2_O_7_ after reaction and **i**–**l** its corresponding EDS elemental maps of **j** Cu, **k** P and **l** mixed elements, respectively, displaying the nanometric Cu/CuPO architecture in both Cu_3_(PO_4_)_2_ and Cu_2_P_2_O_7_.

Based on the theoretical studies, CuPO catalysts were synthesized using a facile and scalable method, as illustrated in the “Methods”, Supplementary Fig. 5 and Note 3. The XRD patterns of the catalyst powders investigated in this study are shown in Fig. 2a, b, with diffraction peaks well indexed to the triclinic phase of Cu_3_(PO_4_)_2_ (PDF#97-006-8811) and the monoclinic Cu_2_P_2_O_7_ (PDF#97-015-7107), respectively, without any impurities. After 1 h (h) of CO_2_RR at a potential of −1.40 V, both Cu_3_(PO_4_)_2_ and Cu_2_P_2_O_7_ are partially reduced to metallic Cu, indicating the coexistence of CuPO and metallic Cu components during CO_2_RR. Furthermore, X-ray absorption fine structure (XAFS) analysis in conjunction with the XRD results proved the coexistence of Cu^2+^ and Cu^0^ species as the CO_2_RR goes on for at least 10 h (Supplementary Figs. 6 and 7). CuO as a control sample was synthesized using the same method, and XRD characterizations were also carried out to identify the material components (Supplementary Fig. 8). The shape and microstructure of Cu_3_(PO_4_)_2_, Cu_2_P_2_O_7_, and CuO were examined via transmission electron microscopy (TEM), and all the three nanoparticles possess irregular shapes (Supplementary Figs. 9–11a). Supplementary Figs. 9–11b give the related high-resolution TEM (HRTEM) images of Cu_3_(PO_4_)_2_, Cu_2_P_2_O_7_, and CuO, and the clear lattice fringes with the spacing of 0.408, 0.316, and 0.252 nm are observed, corresponding to the (110) plane of triclinic phase Cu_3_(PO_4_)_2_, the ($\bar{2}$02) plane of monoclinic phase Cu_2_P_2_O_7_ and the (111) plane of monoclinic phase CuO, respectively. Further high-angle annular dark-field scanning transmission electron microscopy (HAADF-STEM, Supplementary Figs. 9c and 10c) and corresponding energy-dispersive spectroscopy (EDS) maps (Supplementary Fig. 9d–f and Supplementary Fig. 10d–f) verify that Cu, O, and P elements are homogeneously distributed along the Cu_3_(PO_4_)_2_ and Cu_2_P_2_O_7_ nanoparticles. Notably, after CO_2_RR, the HAADF-STEM image of Cu_3_(PO_4_)_2_ shows the agglomeration of small-size nanoparticles (Supplementary Fig. 12), and the HRTEM image exhibits that metallic Cu nanocrystal with a d-spacing of (111) plane is closely contacted to Cu_3_(PO_4_)_2_ with a d-spacing of (210) and (012) plane (Fig. 2c). False color further highlights the close contact between metallic Cu and Cu_3_(PO_4_)_2_ (Supplementary Fig. 13a). As shown in the enlarged HAADF- STEM image (Fig. 2d) and the element mappings (Fig. 2e–g), the segregated Cu nanoparticles are observed, which is in agreement with the XRD result. As for Cu_2_P_2_O_7_, the TEM images show that metallic Cu nanoparticles with sizes ranging from 3 ~ 25 nm that are featured by the d-spacing of (111) planes are distributed on Cu_2_P_2_O_7_ that are featured by the d-spacing of (022) planes after the CO_2_RR process (Fig. 2h and Supplementary Fig. 12b). The distribution of metallic Cu and Cu_2_P_2_O_7_ was further confirmed by the false-color HRTEM (Supplementary Fig. 13b) and HAADF-STEM images (Fig. 2i) overlapped with the corresponding EDS element mappings (Fig. 2j–l). By contrast, the TEM images of the CuO control sample after CO_2_RR show that the pristine CuO phase has been reduced to Cu^0^ during the test (Supplementary Fig. 14).

**Fig. 3:**
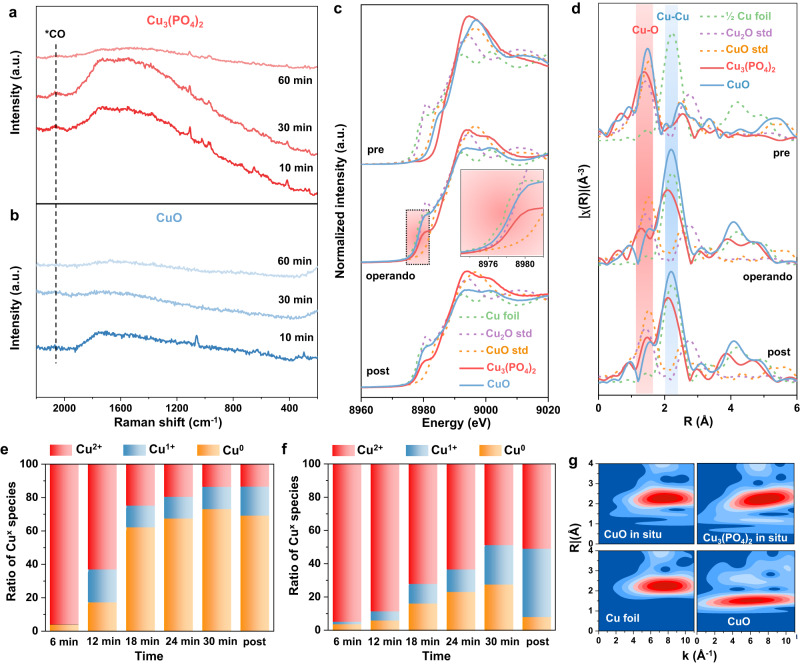
Structural evolution investigation of CuPO and CuO catalysts during CO_2_RR. In situ Raman spectra of **a** Cu_3_(PO_4_)_2_ and **b** CuO catalysts, respectively, during CO_2_RR at −1.40 V in CO_2_-saturated 0.1 M KHCO_3_, suggesting the existence of Cu_3_(PO_4_)_2_ and the disappear of CuO during CO_2_RR. XAFS characterization of **c** the normalized Cu *K*-edge operando XANES spectra, **d** Fourier-transformed Cu *K*-edge EXAFS spectra, and **e**, **f** Calculated ratio of Cu oxidation states in **e** CuO and **f** Cu_3_(PO_4_)_2_ catalysts from linear combination fitting with respect to time during 30 min of reaction at −1.45 V. The in situ Raman spectra along with operando XAFS analysis demonstrate the coexistence of Cu^2+^ and Cu^0^ components in Cu_3_(PO_4_)_2_ and the reductive process of oxide Cu species to the metallic Cu^0^ states in the CuO control sample. **g** Morlet WT of the *k*^3^-weighted operando EXAFS data for the Cu_3_(PO_4_)_2_ and CuO samples with standard Cu foil and CuO powder as controls, suggesting the coexistence of Cu–O and Cu–Cu bond in Cu_3_(PO_4_)_2_ during CO_2_RR.

In order to identify the variation of surface valence states and chemical compositions of CuPO and CuO samples after CO_2_RR, X-ray photoelectron spectroscopy (XPS) was conducted (Supplementary Figs. 15–18). As observed in Supplementary Fig. 15, the peaks detected at about 933.2 and 935.5 eV of initial Cu_2_P_2_O_7_ and CuO can be ascribed to the Cu^2+^ component^33^. After 1 h’s CO_2_RR at −1.40 V, the characteristic Cu^0^/Cu^1+^ (932.5 eV) peak appears and occupies a dominant position in CuO, indicating the surface Cu^2+^ components of the CuO sample are mainly reduced to Cu^0^/Cu^1+^ (ref. ^33^). While the Cu_2_P_2_O_7_ sample continues to exhibit the main component of Cu^2+^ species after CO_2_RR, which benefits from the stability at a negative potential of Cu_2_P_2_O_7_. Due to the fact that the binding energies of the Cu^0^/Cu^1+^ states are difficult to distinguish in the Cu 2p region, we further performed a Cu LMM auger peak analysis (Supplementary Fig. 16). The peaks at around 567.7 and 570.0 eV demonstrate that both Cu^0^ and Cu^1+^ are formed in Cu_2_P_2_O_7_ and CuO after CO_2_RR^34^. Furthermore, the feature around 570.4 eV confirms the dominant existence of Cu(OH)_2_ (ref. ^34^), indicating the component Cu^2+^ species are preserved in the Cu_2_P_2_O_7_ catalyst after CO_2_RR. For the spectrum in the P 2*p* region (Supplementary Fig. 17a), the characteristic P 2*p*_1/2_ and 2*p*_3/2_ peaks for P_2_O_7_^4−^ are detected at the BE of 134.8 and 133.8 eV, indicating the phosphate group persists during the test^35^. Further evidence is derived from the O 1*s* spectrum, the peak located at 531.3 eV can be assigned to the non-bridging O in the phosphate group (P=O), and the peak at the BE of 533.1 eV can be attributed to the symmetric bridging O in P–O–P group (Supplementary Fig. 17b)^35^. Fourier-transformed infrared (FTIR) and Raman spectroscopy are two powerful methods to analyze low-frequency modes in the phosphates system. As shown in the FTIR spectrum of Cu_2_P_2_O_7_, characteristic peaks that appear in 1100–900 cm^−1^ region are attributed to symmetric and asymmetric P–O bonds, as well as the asymmetric P–O–P bridge vibration, confirming the phosphate group in the sample before and after CO_2_RR (Supplementary Fig. 19)^36^. The Raman spectrum of Cu_2_P_2_O_7_ is displayed in Supplementary Fig. 20a, peaks located at the frequency areas of 1250–950 cm^−1^ are known to be assigned to the P–O stretching modes of [P_2_O_7_]^4−^. The symmetric and asymmetric stretch of P–O–P bridge appear in 930–970 and 680–760 cm^−1^ regions respectively, and characteristic peaks observed in the area of 600–500 cm^−1^ (PO_2_^2-^ radical) and 500–370 cm^−1^ (P–O–P bridge) correspond to the P–O–P bending vibration^37^. In addition, the P–O–P deformations, the PO_3_ rocking and deformation modes, and the external and torsional modes are detected in the region of 430–180 cm^−1^ (ref. ^38^). These characteristic peaks are confirmed as pyrophosphate compounds, and they further demonstrate the durability of the pyrophosphate group during CO_2_RR. For comparison, the Raman spectra of CuO are given in Supplementary Fig. 20b, and the bands found at 293, 345, and 633 cm^−1^ are attributed to the standard Ag, Bg (1), and Bg (2) mode, respectively^39^. After being applied at −1.40 V for 1 h, no obvious peak could be detected in the Raman spectra, which proves that the CuO was totally reduced to Cu(0) during CO_2_RR. We then implemented in situ Raman spectroscopy on the Cu_3_(PO_4_)_2_ catalysts, which were measured under a constant potential of −1.40 V in the CO_2_-saturated 0.1 M KHCO_3_ electrolyte. Figure 3a shows the time-dependent in situ Raman spectra of Cu_3_(PO_4_)_2_. The stretching of the PO_4_^3−^ unit is observed at around 1000–1100 cm^−1^, and the bands around 450–650 cm^−1^ are attributed to the bending vibration of the PO_4_^3-^ unit^38^. Considering that a moderate decrease in Raman spectra is observed in 60 minutes (min), we concluded that metallic Cu^0^ formed as the reduction of Cu_3_(PO_4_)_2_ started, whereas residual Cu_3_(PO_4_)_2_ remained present in the bulk. Noticeably, the atop-adsorbed CO (CO_atop_) peak, acting as an important intermediate for the C–C coupling process, was detected at about 2060 cm^−1^ (ref. ^40^). As for the CuO control sample in Fig. 3b, the Raman peaks of CuO disappeared after 30 min, which indicated its entire reduction; moreover, no obvious *CO peak was detected. The in situ Raman measurement result suggests that sufficient coverage of CO* could be built on the Cu/Cu_3_(PO_4_)_2_ catalyst, promoting further dimerization during CO_2_RR.

To explore the oxidation state evolution of the electrocatalysts during CO_2_RR, we carried out operando XAFS measurements under the electrochemical condition at −1.45 V (details in the Methods, Supplementary Fig. 21). The operando X-ray absorption near edge structure (XANES) spectra clearly show that the CuO sample appears metallic Cu^0^ during CO_2_RR and kept its metallic Cu^0^ state after test, while Cu_3_(PO_4_)_2_ stays in higher valence states than metallic Cu(0) in the whole course of CO_2_RR (Fig. 3c). Figure 3d exhibits the operando extended region of the XAS (EXAFS) spectra, and peaks at about 1.5 Å and 2.1 Å are attributed to the coordination of Cu–O and Cu–Cu, respectively^41,42^. A distinct peak of Cu−O coordination can be detected in CuPO at about 1.4 Å, indicating the existence of oxygen-bearing Cu species, and the peak shifts to a smaller radial distance gradually when CO_2_RR takes place, implying the partial reduction of Cu^2+^. The enhancement of peak (Cu−Cu) in the samples (solid lines) suggests the emergence of Cu^0^, and Cu_3_(PO_4_)_2_ catalyst shows a higher Cu–O coordination number and a lower Cu–Cu coordination number compared with CuO indicating the stability of Cu_3_(PO_4_)_2_ at a negative potential to a certain extent. Meanwhile, we fitted the EXAFS data of Cu_3_(PO_4_)_2_ during CO_2_RR, and the fitting results further show that Cu–O coordination and Cu–Cu coordination exist simultaneously in the Cu_3_(PO_4_)_2_ catalyst under reaction conditions (Supplementary Fig. 22 and Table 1). To quantitatively analyze the evolution of oxidation states during CO_2_RR, we processed in situ spectra using an LCF of the CuO and Cu_3_(PO_4_)_2_ samples (Fig. 3e, f). The LCF spectra and data prove that the fitting results are reliable (Supplementary Fig. 23 and Table 2). From the LCF results, we calculated the ratios of Cu oxidation species presented at each 6 min under −1.45 V. After 6 min, the majority of CuO (96%) and Cu_3_(PO_4_)_2_ (95%) were both in the Cu^2+^ oxidation state, while they had respectively decreased to 25% and 72% after a further 12 min, which revealed that the transition between Cu^2+^ and Cu^0^ for CuO was more rapid, while the reduction for Cu_3_(PO_4_)_2_ was slower. After the catalysts had been electrochemically reduced via CO_2_RR for 30 min, the majority of the CuO sample had been almost entirely reduced to Cu^0^ (73%), while the Cu_3_(PO_4_)_2_ sample was still 49% composed of the Cu^2+^. These results show that Cu^2+^ of Cu_3_(PO_4_)_2_ may be stabilized so that Cu^2+^ and Cu^0^ could be coexistent under −1.45 V for over 30 min. Solid support for the coexistent metallic Cu^0^ and Cu^2+^ during the CO_2_RR process in Cu_3_(PO_4_)_2_ was also provided through the additional analysis of the Morlet wavelet transform (WT). As shown in Fig. 3g, a WT maximum at 6–8 Å^−1^ assigned to Cu–Cu bond is visible in the CuO sample during CO_2_RR at −1.45 V, which indicates the metallic Cu^0^ species occupy the main composition in the bulk during the test. By contrast, the Cu_3_(PO_4_)_2_ catalyst exhibits a distinct feature at 5–10 Å^−1^ besides the WT maximum at 6–8 Å^−1^, which represents the coexistence of Cu–O and Cu–Cu bond, demonstrating the component of Cu oxidation species still remained under the same reaction condition^41^. The operando XAFS analysis in conjunction with in situ Raman results clearly proved the specific coexistence of Cu^2+^ and Cu^0^ species in CuPO samples under CO_2_RR condition which could affect the selectivity.

### Electrochemical CO_2_ conversion

**Fig. 4:**
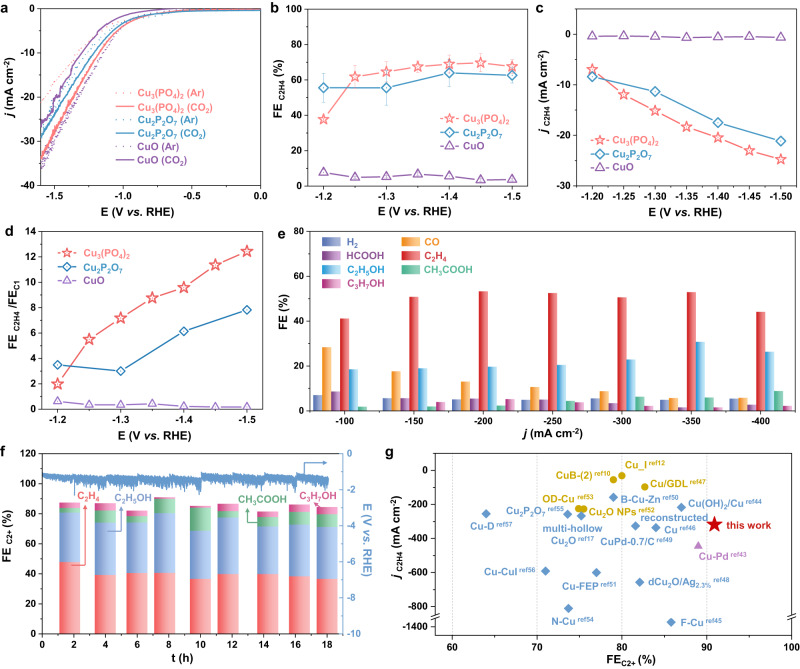
Electrochemical performance evaluation of CuPO and CuO catalysts for CO_2_RR. **a** LSV curves of CuPO/GC and CuO/GC catalysts with respect to RHE at a scan rate of 1 mV/s in the CO_2_- or Ar-saturated 0.1 M KHCO_3_ electrolyte, indicating the occurrence of CO_2_RR. **b** FEs of C_2_H_4_ of catalysts at different applied potentials in CO_2_-saturated 0.1 M KHCO_3_, suggesting the superior C_2_H_4_ selectivity for CuPO. Error bars above were all based on the standard deviation of three measurements at each potential. **c** Potential dependence of *j*_C2H4_ on CuPO/GC and CuO/GC, exhibiting higher C_2_H_4_ activity for CuPO. **d** FE_C2H4_/FE_C1_ ratios of catalysts at different applied potentials, suggesting the superior C_2_H_4_ selectivity for CuPO/GC. **e** FEs for products over Cu_3_(PO_4_)_2_/Cu/PTFE at various applied current densities in a flow cell reactor with 2.0 M KOH as the electrolyte. **f** FEs of various products produced by Cu_3_(PO_4_)_2_/Cu/PTFE at the applied *j* of −300 mA cm^−2^ for 18 h in the 2.0 M KOH electrolyte (refresh electrolyte for every 2 h), and the liquid products were collected after the reaction run for more than 30 min. **g** Maximum FE_C2+_ of Cu_3_(PO_4_)_2_/Cu/PTFE and recently reported Cu-based CO_2_RR catalysts (details in Supplementary Table 5). Blue rhombus: alkaline medium; yellow circle: neutral medium; violet triangle: acidic medium.

The activity of CuPO catalysts for electrocatalytic CO_2_RR was evaluated in an H-cell with CO_2_-saturated 0.1 M KHCO_3_ solution as an electrolyte, and the electrodes were prepared by dropping casting ink solution onto the glassy carbon electrodes (GCE, see more details in the “Methods”). The linear sweep voltammetry (LSV) curves of the Cu_3_(PO_4_)_2_/GC, Cu_2_P_2_O_7_/GC, and CuO/GC in the CO_2_-saturated (full line) or Ar-saturated (dotted line) environments are shown in Fig. 4a. The Cu_3_(PO_4_)_2_ and Cu_2_P_2_O_7_ catalysts exhibited larger geometric reduction *j* in the CO_2_ atmosphere, which demonstrated the reaction priority to CO_2_RR. Thereafter, Cu_3_(PO_4_)_2_/GC, Cu_2_P_2_O_7_/GC, and CuO/GC were measured over a range of potentials for catalytic activity (Supplementary Fig. 24). Cu_3_(PO_4_)_2_/GC shows a marked selectivity for C_2_H_4_ (FE_C2H4_ > 60%) under potentials from −1.25 to −1.50 V, with a topmost FE_C2H4_ reaching 69.7% at −1.45 V (Fig. 4b), corresponding to a *j*_C2H4_ of −23.0 mA cm^−2^, similar to what is tested on Cu_2_P_2_O_7_/GC (FE_C2H4_ of 64.0% and *j*_C2H4_ of −17.6 mA cm^−2^ at −1.40 V (Fig. 4c). As a comparison, the CuO/GC is less selective and its CO_2_RR catalysis yields CO (<8% FE) and CH_4_ (<18% FE), as well as C_2_H_4_ (<8% FE) in −1.20 ~ −1.50 V (Fig. 4c and Supplementary Fig. 24). In particular, the ratios of C_2_H_4_ and C_1_ products for Cu_3_(PO_4_)_2_/GC and Cu_2_P_2_O_7_/GC reach about 12.4 and 7.8 at −1.5 V, which is overwhelmingly superior to CuO/GC (Fig. 4d). Specific values for all products are given in Supplementary Tables 3 and 4.

To further improve the CO_2_RR current density, we constructed gas diffusion electrodes (GDE) with the Cu_3_(PO_4_)_2_ catalyst deposited on the Cu-coated polytetrafluoroethylene gas diffusion layers (Cu_3_(PO_4_)_2_/Cu/PTFE) and evaluated CO_2_RR performance utilizing a flow cell (details in the “Methods”). The 2.0 M KOH alkaline electrolyte was used to enhance the conductivity and improve the CO_2_RR kinetics by suppressing H_2_ evolution. As shown in Fig. 4e, the Cu_3_(PO_4_)_2_/Cu/PTFE catalyst was screened at different current densities and reached a maximum FE_C2+_ of 90.9% (52.8% ethylene, 30.7% ethanol, 5.9% acetic acid, and 1.5% n-propyl alcohol) with *j*_c2+_ of −318.2 mA cm^−2^ corresponding to experiments performed at *j*_tot_ = −350 mA cm^−2^. Supplementary Fig. 25 exhibits the chronopotential curves obtained at different *j*. Meanwhile, the bare Cu/PTFE showed FE_C2+_ of 58.9 ~ 77.2% with the *j*_tot_ ranging from −100 to −400 mA cm^−2^ (Supplementary Fig. 26), proving the Cu_3_(PO_4_)_2_ catalyst has excellent CO_2_-to-C_2+_ conversion performance intrinsically. Electrochemical stability is also a key parameter for CO_2_RR performance. Remarkably, Cu_3_(PO_4_)_2_/Cu/PTFE unveils a desirable stability in the flow cell during CO_2_RR for 18 h (Fig. 4f). The total current density keeps at −300 mA cm^−2^, and FE_C2+_ remains above 80% after 18 h of reaction, representing that the catalyst is stable during CO_2_RR. Furthermore, we compared the maximum FE_C2+_ of 90.9% for Cu_3_(PO_4_)_2_/Cu/PTFE with that for other Cu-based electrocatalysts (Fig. 4g and Supplementary Table 5), which was found to possess a remarkable C_2+_ production selectivity compared to the reported Cu-based catalysts^10,12,17,43–57^.

### Insights into CO_2_-to-C_2+_ electroreduction

**Fig. 5:**
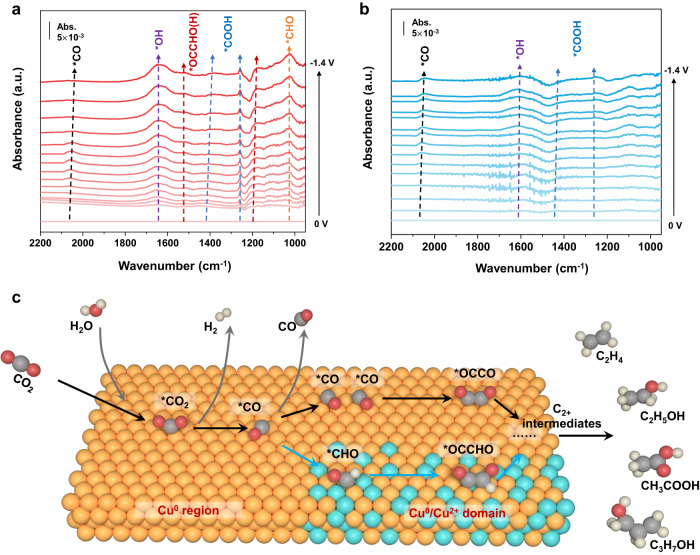
Insights into CO_2_-to-C_2+_ electroreduction. In situ SEIRAS differential spectra of the **a** Cu_3_(PO_4_)_2_ and **b** CuO sample in CO_2_ purged 0.1 M KHCO_3_ electrolyte in real-time condition. **c** Schematic illustration on proposed role of Cu(II)/Cu(0) interfaces in CO_2_-to-C_2+_ conversion. Red: oxygen; gray: carbon; white: hydrogen; origin: Cu^0^; golden: Cu^2+^.

The CO_2_RR intermediates chemisorbed on CuPO and CuO were assessed via SEIRAS to determine the mechanism for boosted C_2+_ selectivity (details in the Methods, Supplementary Fig. 27). As shown in Fig. 5a, the in situ SEIRAS differential spectra of Cu_3_(PO_4_)_2_ exhibit peaks at about 2065 cm^−1^ and 1025 cm^−1^, which are associated with the absorbed *CO and the nonplanar vibration (O=C–H) of *CHO on the catalyst surface, respectively^58,59^. Additionally, the peaks detected at about 1255 and 1385–1410 cm^−1^ could be attributed to the C–O stretch and symmetric vibration (vibration of O–C=O) of *COOH, respectively^59^. Prominently, based on reported experimental and theoretical studies, the distinctive 1180 and 1520 cm^−1^ peaks can demonstrate the existence of *OCCHO intermediate; it increased on scanning to more negative potentials, which is consistent with the trend of C_2+_ products’ formation rates^59–61^. To support the band assignments of the IR to *OCCHO theoretically, we simulated the IR of *OCCHO intermediates that adsorbed at the Cu^0^/Cu^2+^ (Cu_3_(PO_4_)_2_) interface. As shown in Supplementary Fig. 28, a band peak with strong oscillator strength is located at 1189 cm^−1^, which is close to our experiment result (1180 cm^−1^) for *OCCHO. Moreover, the peak intensity of *CO peaks on Cu_3_(PO_4_)_2_ enhances first as *CO increases, and then gradually decreases as the applied potential continues to turn negative, suggesting the *CO is consumed as dimerization accelerates. In comparison, the absorbed *CO continues to accumulate and the C–C coupling bond is not detected on the CuO sample, which would be entirely reduced to Cu^0^ during CO_2_ reduction (Fig. 5b). As illustrated in the schematic diagram (Fig. 5c), the in situ SEIRAS study in conjunction with theoretical research reveals that the stable and abundant Cu^0^/Cu^2+^ interfaces derived from Cu phosphate-based electrocatalysts can facilitate the pathway of *CHO coupling with *CO to form *OCCHO, thus improving the selectivities of C_2+_ products during CO_2_ reduction.

## Discussion

In summary, we have theoretically predicted and experimentally constructed the in situ formation of nanometric Cu/CuPO with rich and stable Cu^0+^/Cu^2+^ interfaces to be the efficient electrocatalyst for highly selective CO_2_-to-C_2+_ products conversion. The resultant nanometric Cu/CuPO could obtain a high FE_C2H4_ of 69.7% in a neutral medium and perform a maximum FE_C2+_ above 90% with industrially relevant current densities in a flow cell configuration. The Cu^2+^ sites of Cu^0+^/Cu^2+^ interfaces were revealed to be available for the formation of *CHO, which then facilely coupled with *CO on the adjacent Cu^0^ surface to form *OCCHO, leading to high-efficiency CO_2_-to-C_2+_ conversion performance. Our work highlights the role of the peculiar Cu^0^/Cu^2+^ interfaces in promoting selectivity toward C_2+_ products, and we believe that this finding will contribute to the design of improved Cu-based electrocatalysts for future CO_2_RR, with high selectivity and underlying mechanisms for its conversion to valuable products.

## Methods

### DFT calculation

All the calculations were performed using the Vienna Ab-initio Simulation Package (VASP) package^62–64^. The exchange-correlation functional was described by the Perdew–Burke–Ernzerhof (PBE) functional^65^ within the generalized gradient approximation (GGA)^66^. The project-augmented wave (PAW) method^67^ was employed to treat core electrons, and the cut-off energy of the plane-wave basis was set to 450 eV. A vacuum layer of 15 Å was applied to separate each periodic unit cell. In the structural optimizations, the Brillouin zone was sampled by 2 × 2 × 1 Monkhorst–Pack mesh k-points, and the bottom two layers of the slab were fixed, and the top three layers and adsorbates were fully relaxed. The empirical correction in Grimme’s scheme was used to describe the van der Waals interactions^68^. Considering the solvation environment, we constructed an explicit solvation model with one layer H_2_O molecules in calculation models.

### Catalyst preparation

For Cu_3_(PO_4_)_2_, 12 mmol Cu(NO_3_)_2_·3H_2_O and 8 mmol NH_4_H_2_PO_4_ were dissolved in H_2_O and stirred well to form a suspension in a beaker. Then, added 6 mmol C_6_H_10_O_8_ to beaker and stirred until the suspension was clear. The beaker was completely covered with tin foil, with some small holes punched in the top, then placed in the oven and dried at 120 °C. Finally, the dried material was transferred to a crucible and calcined at 700 °C for 1 h in a muffle furnace at a heating rate of 5 °C/min. The preparation method of Cu_2_P_2_O_7_ was the same as the above procedure except for changing the mass of Cu(NO_3_)_2_·3H_2_O to 8.5 mmol. The preparation method of CuO was the same as that of Cu_3_(PO_4_)_2_ except that NH_4_H_2_PO_4_ was not added. All reagents were commercially available as analytical grade (Supplementary Note 3).

### Working electrodes preparation

For the Cu_3_(PO_4_)_2_/CP, Cu_2_P_2_O_7_/CP, and CuO/CP used for XRD, XPS, Raman, FTIR, and XAFS measurements. The sample ink was prepared by dispersing 10 mg of catalyst (Cu_3_(PO_4_)_2_, Cu_2_P_2_O_7_, or CuO) and 100 μL of Nafion solution into 0.5 mL of H_2_O and 1.5 mL of iso-propanol, followed by sonication for more than 1 h. The sample ink was sprayed on carbon paper using an air-brush, and the total catalyst loading was about 2.0 mg cm^−2^. For the GCE working electrode used in H-Cell electrochemical measurements, the sample ink was prepared by dispersing 5 mg catalyst (Cu_3_(PO_4_)_2_, Cu_2_P_2_O_7_ or CuO) and 50 μL Nafion solution into 0.125 mL H_2_O and 0.375 mL iso-propanol followed by sonication for more than 30 min. 2.5 μL ink was dropped on GCE (with a diameter of 3 mm) and dried in the air for 2 times, with the total loading amount of catalyst about 0.7 mg cm^−2^.

For Cu/PTFE, bare PTFE with an average pore diameter of 0.22 μm was used. Approximately 600 nm nominal thick Cu films were constructed by vacuum evaporation method on the PTFE substrate using Cu target material (99.999%) at an evaporation rate of around 0.5 Å s^−1^ in an OMV FS300-S6 evaporating tool at a base pressure of <6*10^−4^ Torr. For Cu_3_(PO_4_)_2_/Cu/PTFE, the sample ink was prepared as above mentioned on carbon paper and sprayed on the Cu/PTFE films electrodes using air-brush, with the total loading amount of catalyst about 1.0 mg cm^−2^.

### Materials characterizations

The crystal structure was determined using X-ray diffraction (Bruker D8 Advanced Diffractometer with Cu Kα radiation). The morphology and structure were characterized by scanning electron microscope (Hitachi S4800) and transmission electron microscopy (TEM, JEOL JEM 2010, operated at 200 kV). Scanning transmission electron microscopy (STEM) characterization was performed using a ThermoFisher Talos F200X. High-angle annular dark-field (HAADF)-STEM images were recorded using a convergence semi angle of 11 mrad, and inner- and outer collection angles of 59 and 200 mrad, respectively. Energy-dispersive X-ray spectroscopy (EDS) was carried out using 4 in-column Super-X detectors. The chemical state was analyzed by X-ray photoelectron spectroscopy (XPS, Thermo Escalab 250), and the binding energy of C 1*s* peak at 284.8 eV was taken as an internal standard. Raman analysis was carried out using a Leica DMLM microscope (Renishaw) with the 514 nm laser. FTIR spectroscopy was characterized on a Nicolet 6700 spectrometer with a spectral range of 4000–400 cm^−1^. XAFS spectra at the Cu K-edge were performed on the 1W1B beamline station of the Beijing Synchrotron Radiation Facility (BSRF), China. Cu foil, Cu_2_O and CuO were used as references. LCF were processed using the ATHENA module implemented in the IFEFFIT software packages.

The in situ XAS measurement was performed in a homemade plastic electrolytic cell. The graphite rod (spectral purity, 3 mm in diameter) and the Ag/AgCl electrode acted as the counter electrode and the reference electrode in the three-electrode system, respectively. The carbon paper coated with specific catalysts was used for the working electrode, and a gas inlet for purging CO_2_ into the electrolyte (0.1 M KHCO_3_) was also contained in the cell. A window (1.1 cm × 1.8 cm) was designed on the cell for the working electrode that enabled XAS measurement under a sensitive fluorescence model while the working potentials were applied. A spectrum can be acquired on average every 1.5 min, and the in situ curves of the samples in Fig. 3c, d were taken at around 36 min of reaction.

The in situ Raman measurement was performed on the Raman spectrometer (LabRAM HR) utilizing an excitation laser with a wavelength of 514 nm and a 50× microscope objective with a numerical aperture of 0.5, 10.6 mm. Before the experiments, calibration was carried out based on the peak at 520 cm^−1^ of a silicon wafer standard. To acquire information about the electroreduction process of Cu_3_(PO)_4_ and CuO, we utilize a spectroelectrochemical cell and detect the in situ Raman of the cathode GDE through a quartz window. The GDEs of Cu_3_(PO)_4_ and CuO were taken as the working electrodes, which were prepared by the catalyst sprayed on carbon paper. Platinum wire and Ag/AgCl were used as the counter electrode and reference electrode, respectively. During the in situ experiment, a peristaltic pump was used to control the flow rate of CO_2_-saturated 0.1 M KHCO_3_ electrolyte at 10 mL/min, while the flow rate of CO_2_ was kept at 10 sccm with a mass flow controller.

In situ surface-enhanced infrared absorption spectroscopy (SEIRAS) was recorded in a homemade reflection accessory with internal reflection configuration using an FTIR spectrometer equipped with a PerkinElmer spectrum 100 detector. FTIR spectra were obtained by averaging 16 scans with a resolution of 8 cm^−1^ at the selected potential. Every spectrum was obtained by applying a single potential step compared to the reference potential (0 V). For the spectroelectrochemical measurements, a thin Au film with a thickness of ∼10 nm is prepared on a Si prism using electroless deposition^69^. The sample ink was prepared as mentioned above on GCE. 25 μL ink was dropped on Au underlayer and dried in the air for 2 times. The catalyst-coated Si crystal was placed in a three-electrode spectroelectrochemical cell as the working electrode, and the counter and reference electrodes were platinum wire and Ag/AgCl electrode (3.5 M KCl), respectively. A gas inlet for purging CO_2_ was also contained in the cell. Electrolyte (0.1 M KHCO_3_) was injected into the cell and gassed with high-purity CO_2_ (99.9999%) prior to electrochemical measurements. A CHI1242C potentiostat was used to record electrochemical responses. Spectra were expressed as absorbance, with positive and negative peaks showing an increase and decrease in signal, respectively.

### Electrochemical measurements

All electrochemical studies were performed using an electrochemical station (CHI 660E). The H-type gas-tight reactor consisted of two compartments separated by a Nafion 117 membrane. In the three-electrode system, a custom-made GCE was used as working electrode with a surface area of 0.07 cm^2^ and a catalyst mass loading of 0.7 mg cm^−2^. Ag/AgCl electrode (3.5 M KCl) and platinum mesh were used as reference electrode and counter electrode, respectively. CO_2_-saturated 0.1 M KHCO_3_ (pH ≈ 6.75) was used as electrolyte. All measured potentials were calibrated to the RHE reference scale using *E*_RHE_ = *E*_Ag/AgCl_ + 0.059 × pH + 0.205 (all potentials were not *iR*-corrected if not mentioned). Linear sweep voltammetry (LSV) at a scan rate of 1 mV s^−1^ were performed in Ar and CO_2_-saturated 0.1 M aqueous KHCO_3_. The current density was calculated by normalizing the current to the corresponding geometric surface area.

The electrocatalytic performance of Cu_3_(PO_4_)_2_ was determined in a flow cell configuration. The device consists of Cu_3_(PO_4_)_2_/Cu/PTFE as the working electrode, nickel foam as the anode and an anion exchange membrane. They were assembled using PTFE gaskets with 2.0 M KOH as the liquid electrolyte flowing (10 mL per min) in the chambers between membrane and working electrode, and membrane and anode. CO_2_ gas was flowed behind the PTFE layer at a rate of 20 sccm, which was decreased to 19 sccm at the outlet due to the consumption of the alkaline electrolyte. Chronopotentiometry experiments were performed at currents of −100 mA cm^−2^, −150 mA cm^−2^, −200 mA cm^−2^, −250 mA cm^−2^, −300 mA cm^−2^, −350 mA cm^−2^, and −400 mA cm^−2^, showing consistent gas product distributions. The liquid products were collected after the reaction run for more than 30 min.

### Products analysis

The gas products of CO_2_ electroreduction were analyzed by gas chromatography (GC online test, RAMIN, GC2060), equipped with a flame ionization detector (FID to detect CO, CH_4_ and C_2_H_4_) and a thermal conductivity detector (TCD to detect H_2_). Ar was used as a carrier. CO_2_ was continuously sparged through the electrolyte (30 mL of catholyte and anolyte in each compartment) at a rate of 20 sccm and was routed into the gas chromatograph. The Faradic efficiencies were tested online and averaged for multiple data.

The liquid products were quantified by ^1^H nuclear magnetic resonance (NMR) (Varian 700 MHz spectrometer, 16.4 T). In a typical analysis, the mixture of 500 mg of the electrolyte and 100 mg of 149 ppm DMSO (used as internal standard) in D_2_O solution was used as measured sample after 2 h of CO_2_RR with *j* applied at −300 mA cm^−2^. The ^1^H spectra were obtained by suppressing the water peak using the pre-saturation method.

Assuming that 12 electrons are required to generate one C_2_H_4_ molecule, the FE and the partial current densities of C_2_H_4_ formation were calculated as below:

1 $${{{{{\rm{FE}}}}}}=\frac{12{FvG}{P}_{0}}{{i}_{{{{{\rm{total}}}}}}{RT}}\times 100\%$$

where *v* = volume concentration of C_2_H_4_ obtained from gas chromatography (GC) data, *p*_*0*_ = 1.013 bar and *T* = 298.15 K, *G* = 20 sccm is the CO_2_ flow rate (19 sccm for 2 M KOH in a flow cell), *i*_total_ (mA) = steady-state cell current, *F* = 96485 C mol^−1^, *R* = 8.314 J mol^−1^ K^−1^. Then:

2 $${j}_{{{{{{{\rm{C}}}}}}_{2}{{{{{\rm{H}}}}}}}_{4}}={{{{{{\rm{FE}}}}}}}_{{{{{{{{\rm{C}}}}}}}_{2}{{{{{\rm{H}}}}}}}_{4}}\times {i}_{{{{{\rm{total}}}}}}\times {({{{{{{\rm{electrode}}}}}}}\,{{{{{\rm{area}}}}}})}^{-1}$$

### Supplementary information

Supplementary Information

Peer Review File

### Source data

Source Data

## Data Availability

Source data are provided with this paper.
