# Supplementary material for: Direct OC-CHO coupling towards highly C2+ products selective electroreduction over stable Cu0/Cu2+ interface
Source: Nat Commun. 2023 Nov 24;14:7681. doi: 10.1038/s41467-023-43182-6 (PMC10667242; doi:10.1038/s41467-023-43182-6)
Supplement: Supplementary file 1 — Supplementary Information [file 41467_2023_43182_MOESM1_ESM.pdf]

# Supplementary Information

## Direct OC-CHO coupling towards highly $C_{2+}$ products selective electroreduction over stable $Cu^0/Cu^{2+}$ interface

Xin Yu Zhang,[1†] Zhen Xin Lou,[1†] Jiacheng Chen,[2] Yuanwei Liu,[1] Xuefeng Wu,[1] Jia Yue Zhao,[1] Hai Yang Yuan,[1*] Minghui Zhu,[2] Sheng Dai,[3] Hai Feng Wang,[4] Chenghua Sun,[5] Peng Fei Liu[1*] & Hua Gui Yang[1*]

[1] Key Laboratory for Ultrafine Materials of Ministry of Education, Shanghai Engineering Research Center of Hierarchical Nanomaterials, School of Materials Science and Engineering, East China University of Science and Technology, 130 Meilong Road, Shanghai, 200237 (China)

[2] State Key Laboratory of Chemical Engineering, School of Chemical Engineering, East China University of Science and Technology, 130 Meilong Road, Shanghai, 200237 (China)

[3] Key Laboratory for Advanced Materials and Feringa Nobel Prize Scientist Joint Research Center, Institute of Fine Chemicals, School of Chemistry and Molecular Engineering, East China University of Science and Technology, 130 Meilong Road, Shanghai, 200237 (China)

[4] Key Laboratory for Advanced Materials, Centre for Computational Chemistry and Research Institute of Industrial Catalysis, School of Chemistry and Molecular Engineering, East China University of Science and Technology, 130 Meilong Road, Shanghai, 200237 (China)

[5] Department of Chemistry and Biotechnology, and Center for Translational Atomaterials, Swinburne University of Technology, Hawthorn, VIC, 3122 (Australia)

[†] These authors contributed equally to this work.

[*] Emails: hgyang@ecust.edu.cn; pfliu@ecust.edu.cn; hyyuan@ecust.edu.cn

This Supplementary Information file includes: Supplementary Figs. 1-28, Supplementary Tables 1-5, Supplementary Notes 1-3, and Supplementary References.

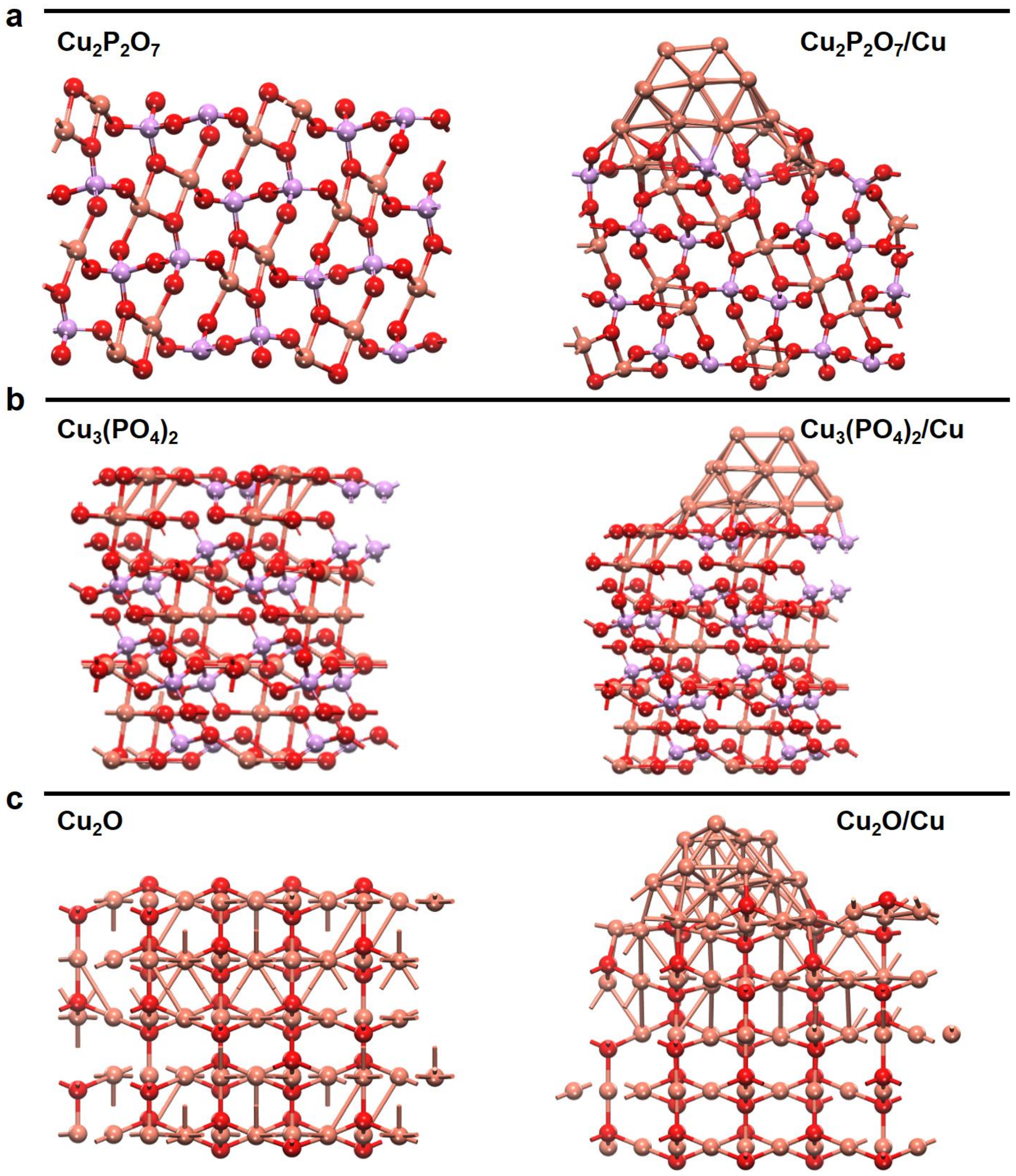


**Supplementary Fig. 1 a-b** $Cu^0/Cu^{2+}$ structure constructed by Cu loaded on a $Cu_2P_2O_7$ and **b** $Cu_3(PO_4)_2$; **c** $Cu^0/Cu^{1+}$ structure constructed by Cu loaded on $Cu_2O$.

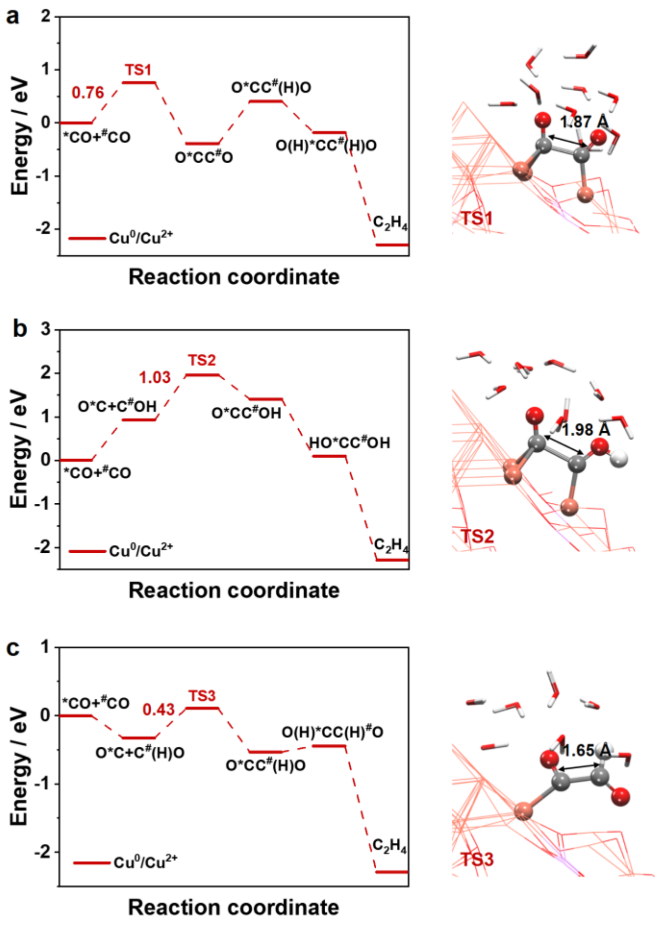


**Supplementary Fig. 2 a-c** Energy profiles of different C-C coupling processes (* and # represent $Cu^0$ and $Cu^{2+}$ sites, respectively), **a** CO-CO, **b** COH-CO, and **c** CHO-CO at the $Cu^0/Cu^{2+}$ interfaces constructed by $Cu_3(PO_4)_2$, and the related transition state structures (TS1~TS3) of different C-C coupling processes.

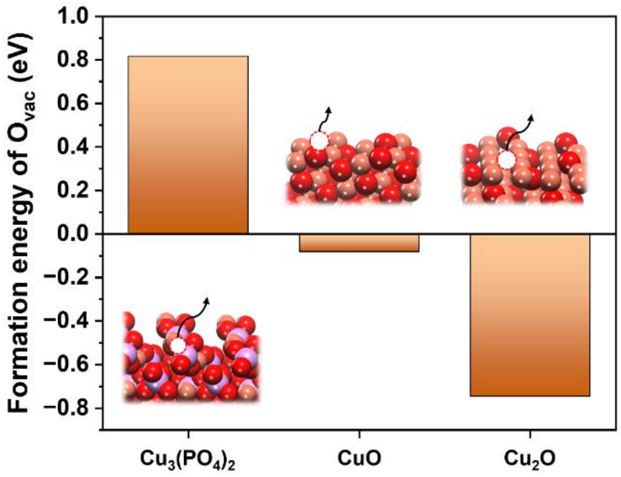


**Supplementary Fig. 3** Formation energy of oxygen vacancies ($E_f(O_{vac})$) on $Cu_3(PO_4)_2$ ($Cu^{2+}$), CuO ($Cu^{2+}$), and $Cu_2O$ ($Cu^{1+}$), $E_f(O_{vac}) = E_{sur} - E_{vac} - (E_{H2O} - E_{H2})$, where $E_{sur}$ and $E_{vac}$ are the energies of the pristine surface and the surface with oxygen vacancy. $E_{H2O}$ and $E_{H2}$ are the energies of $H_2O$ and $H_2$ molecules. Both CuO (-0.08 eV) and $Cu_2O$ (-0.75 eV) have negative formation energies of $O_{vac}$, implying that the lattice O on their surfaces is relatively easy to dissolve. By comparison, the formation energy of the $O_{vac}$ on the surface of $Cu_3(PO_4)_2$ is 0.82 eV, meaning that the O atoms on $Cu_3(PO_4)_2$ can remain relatively stable on the surface.

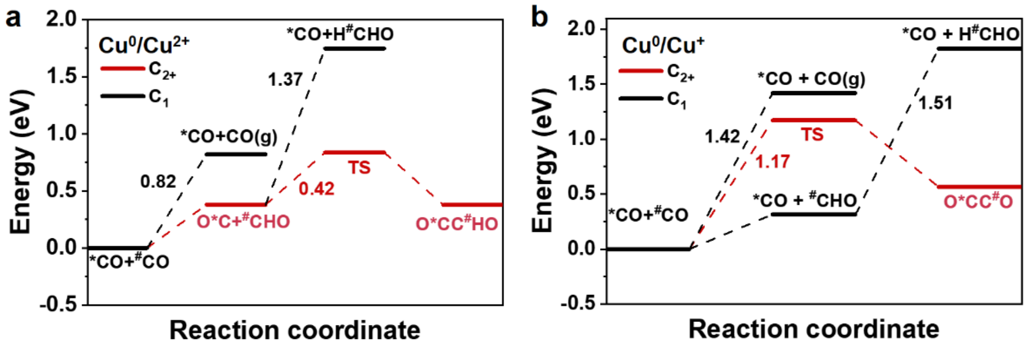


**Supplementary Fig. 4 a-b** Competitive reaction of commonly reported $C_1$ paths (desorption of CO and hydrogenation of CHO to HCHO) and most likely $C_{2+}$ paths on **a** $Cu^0/Cu^{1+}$ (*CO + #CO → OC-CO) and **b** $Cu^0/Cu^{2+}$ (*CO + #CHO → OC-CHO), * and # represent $Cu^0$ and $Cu^{2(1)+}$ sites, respectively. On $Cu^0/Cu^{2+}$, the direct desorption of CO from $Cu^{2+}$ site requires 0.82 eV, while further hydrogenation of CO (formed *CHO) requires only 0.38 eV; the subsequent C-C coupling process overcomes a low energy barrier ($E_a$) of only 0.46 eV, which is much less than the further hydrogenation of CHO to HCHO ($E_a$ = 1.37 eV). On $Cu^0/Cu^{1+}$, the direct desorption of CO on $Cu^{1+}$ site requires 1.42 eV; although the CO hydrogenation to CHO is relatively easy, the further CHO hydrogenation to HCHO needs to cost energy of 1.51 eV. It is obvious that both the CO desorption and CHO further hydrogenation are more difficult to occur compared with the most favored C-C coupling step on $Cu^0/Cu^{1+}$ and $Cu^0/Cu^{2+}$. From the above results, it can be expected that for both $Cu^0/Cu^{1+}$ and $Cu^0/Cu^{2+}$, the selectivity for the $C_{2+}$ products could be more favorable than that for $C_1$ products.

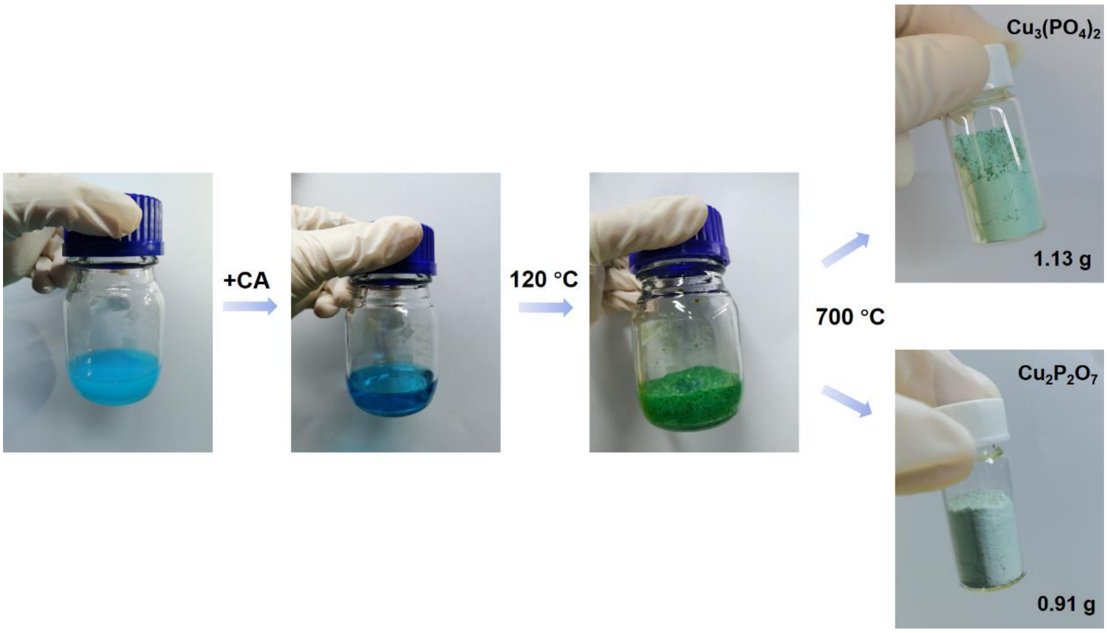


**Supplementary Fig. 5** Schematic illustration of the sol-gel preparation process, showing that the final $Cu_3(PO_4)_2$ (~1.13 g) and $Cu_2P_2O_7$ (~0.91 g) products were obtained.

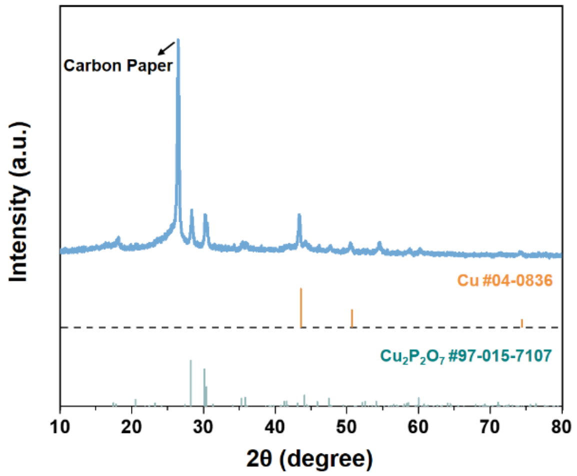


**Supplementary Fig. 6** XRD pattern of $Cu_2P_2O_7$ coated on carbon paper after $CO_2$RR at the applied potential of -1.40 V in $CO_2$-saturared 0.1 M $KHCO_3$ for 10 hours.

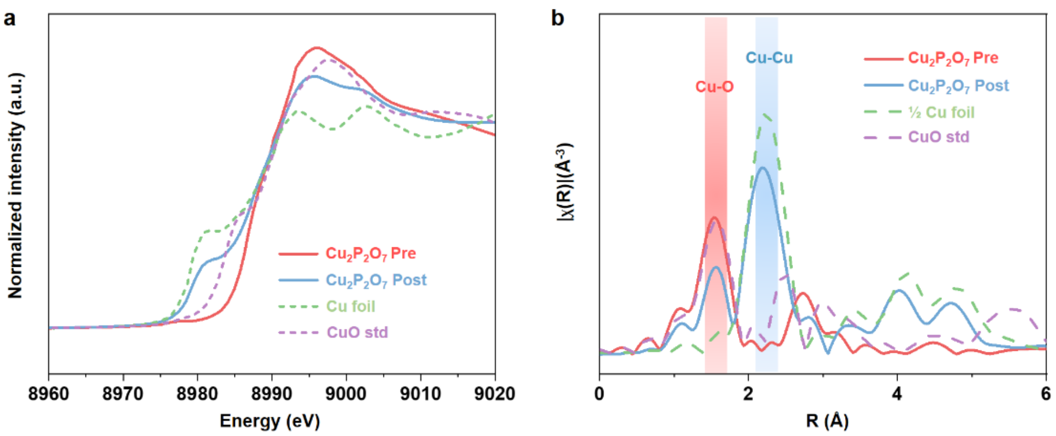


**Supplementary Fig. 7** (a) Normalized Cu *K*-edge XANES spectra and (b) Fourier-transformed Cu *K*-edge EXAFS spectra of $Cu_2P_2O_7$ before and after 10 hour's reaction at the applied potential of -1.40 V in $CO_2$-saturared 0.1 M $KHCO_3$.

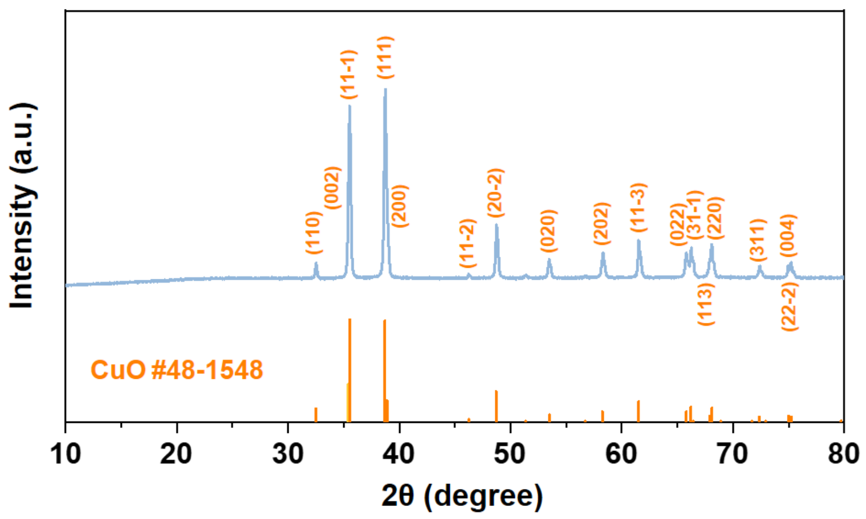


**Supplementary Fig. 8** XRD pattern of CuO control sample, which indicates the crystalline phase of CuO.

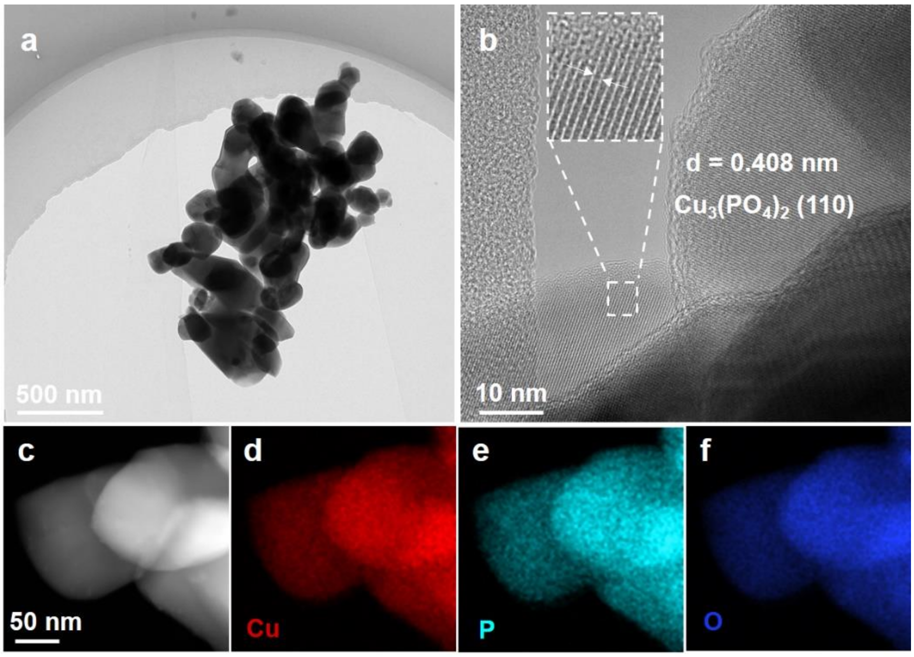


**Supplementary Fig. 9** **a** TEM image and **b** HRTEM image of the $Cu_3(PO_4)_2$ sample. **c** HAADF-STEM image of $Cu_3(PO_4)_2$ and **d-f** corresponding elemental maps of **d** Cu, **e** P, and **f** O elements, respectively.

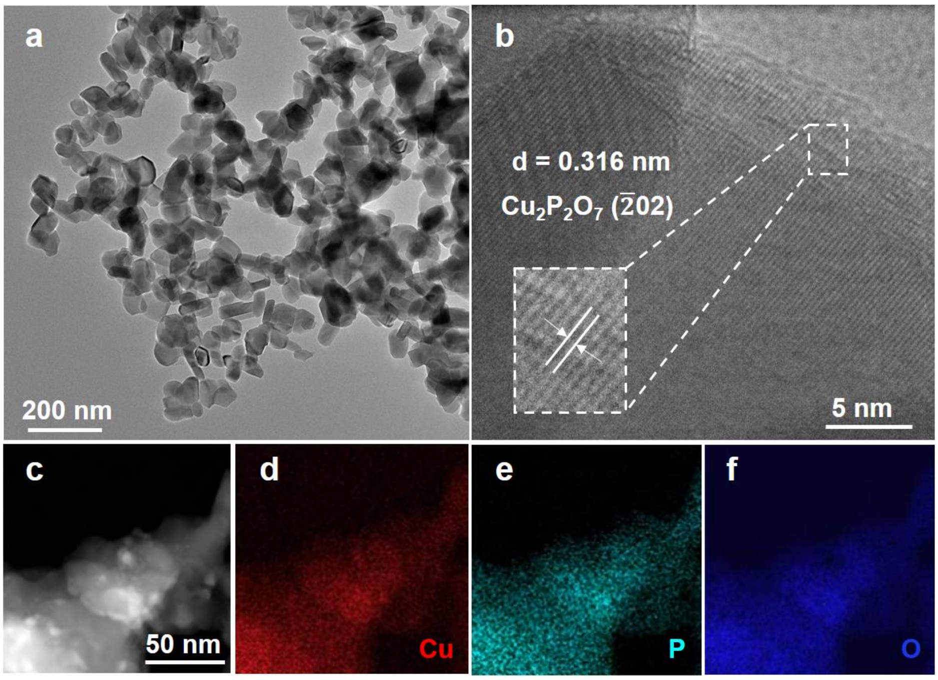


**Supplementary Fig. 10** **a** Low-magnificaiton TEM image and **b** HRTEM image of the $Cu_2P_2O_7$ sample. **c** HAADF-STEM image of $Cu_2P_2O_7$ and **d**-**f** cooresponding elemental maps of **d** Cu, **e** P, and **f** O elements, respectively.

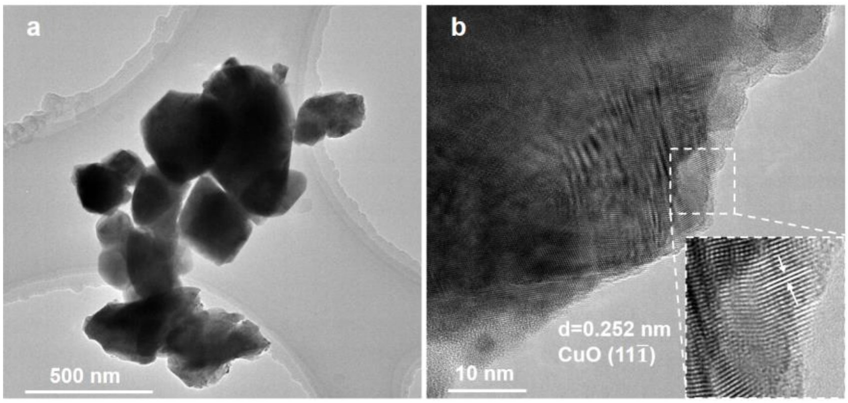


**Supplementary Fig. 11 a** TEM image and **b** HRTEM image of the CuO sample.

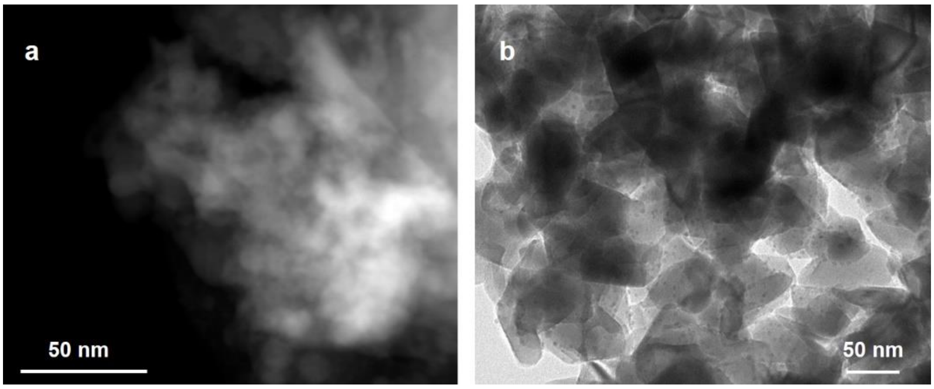


**Supplementary Fig. 12 a** HAADF-STEM image of the $Cu_3(PO_4)_2$ sample and **b** TEM image of the $Cu_2P_2O_7$ sample after 0.5 hour's $CO_2$RR at the applied potential of -1.40 V *vs*. RHE.

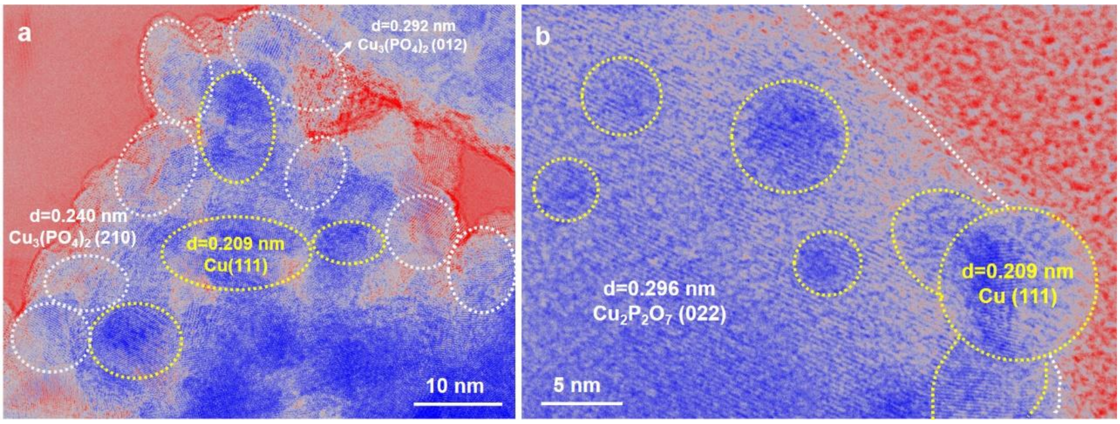


**Supplementary Fig. 13** False-color HRTEM images of the **a** $Cu_3(PO_4)_2$ and **b** $Cu_2P_2O_7$ sample after 0.5 hour's $CO_2$RR at the potential of -1.40 V *vs*. RHE.

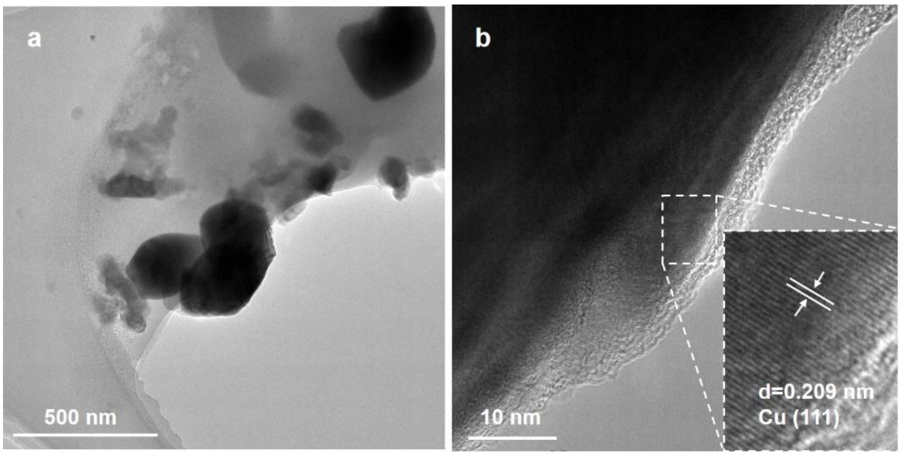


**Supplementary Fig. 14 a** TEM image and **b** HRTEM image of the CuO control sample after 0.5 hour's $CO_2$RR at the applied potential of -1.40 V *vs*. RHE, showing that the pristine CuO phase has been reduced to metallic Cu during the test.

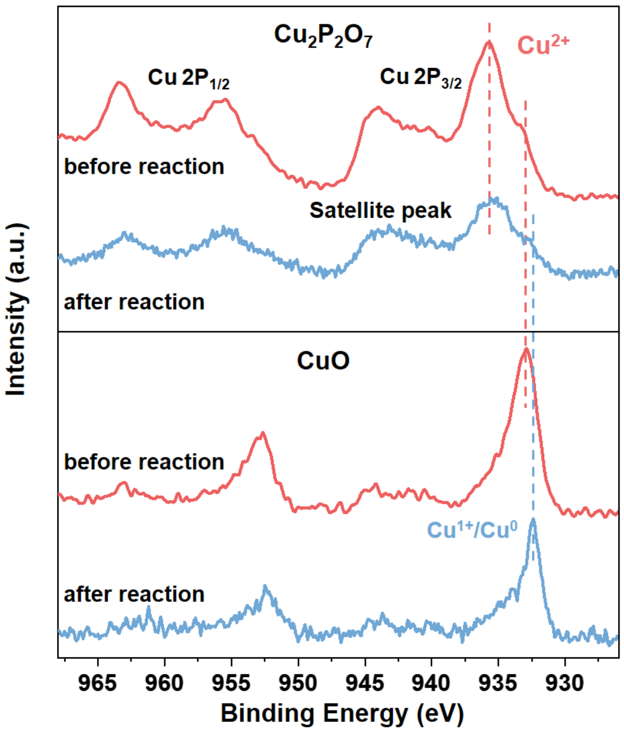


**Supplementary Fig. 15** XPS spectra of Cu 2p region of $Cu_2P_2O_7$ and CuO control sample collected before and after $CO_2RR$ at -1.40 V *vs*. RHE for 1 h, showing that the Cu species of $Cu_2P_2O_7$ exhibited the main component of $Cu^{2+}$, while Cu species of CuO were largely reduced to metallic $Cu^0$ states after $CO_2RR$.

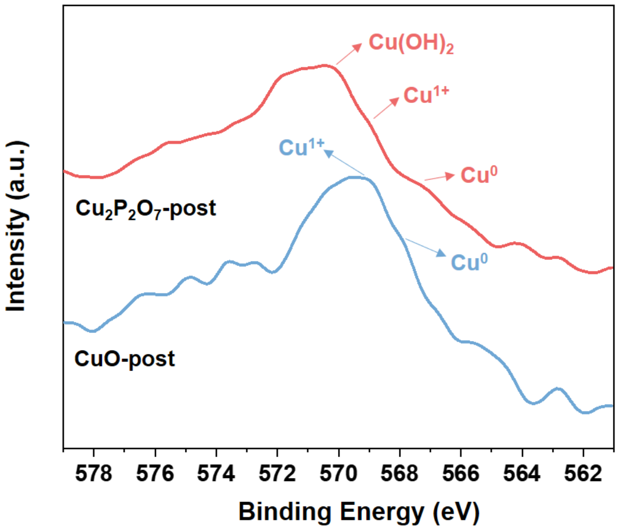


**Supplementary Fig. 16** Cu LMM Auger spectra of the $Cu_2P_2O_7$ and CuO control sample measured after 1 h of $CO_2$RR at -1.40 V *vs*. RHE.

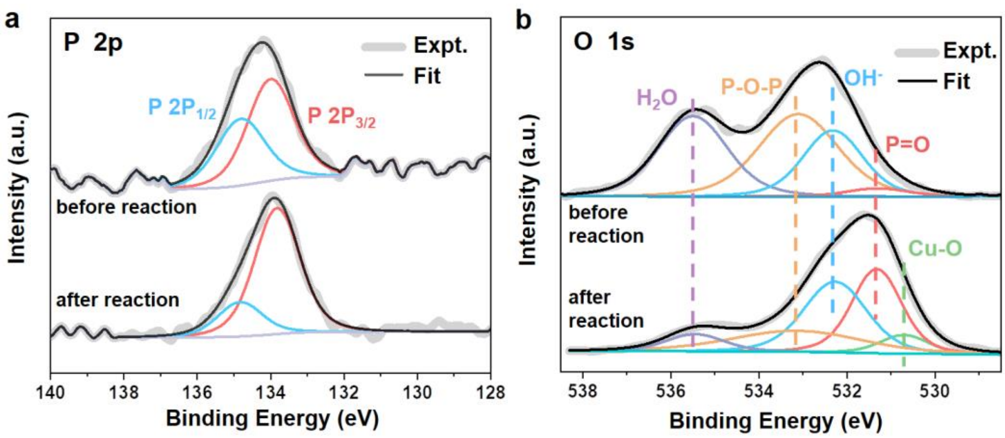


**Supplementary Fig. 17 a**, **b** XPS spectra of $Cu_2P_2O_7$ before and after 1 hour's $CO_2RR$ at the applied potential of -1.40 V *vs*. RHE in **a** P 2p and **b** O 1s regions, indicating the phosphate group persisted during the test. Notes: Cu-O might result from the slight oxidation in the air.

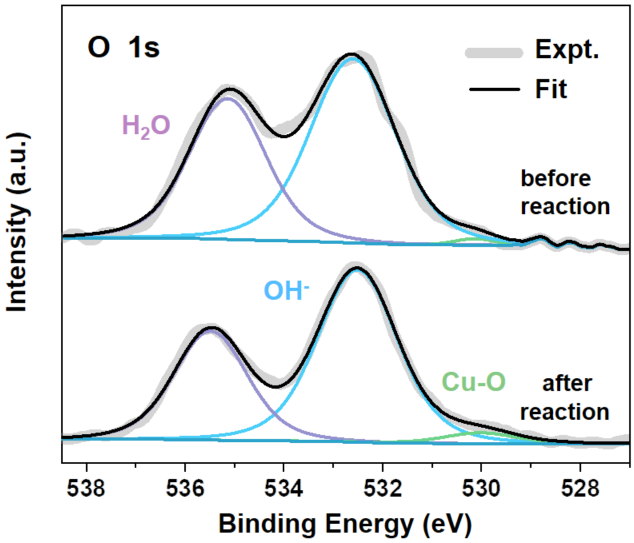


**Supplementary Fig. 18** XPS spectra of CuO control sample before and after 1 hour's $CO_2$RR at the applied potential of -1.40 V *vs*. RHE in O 1s region.

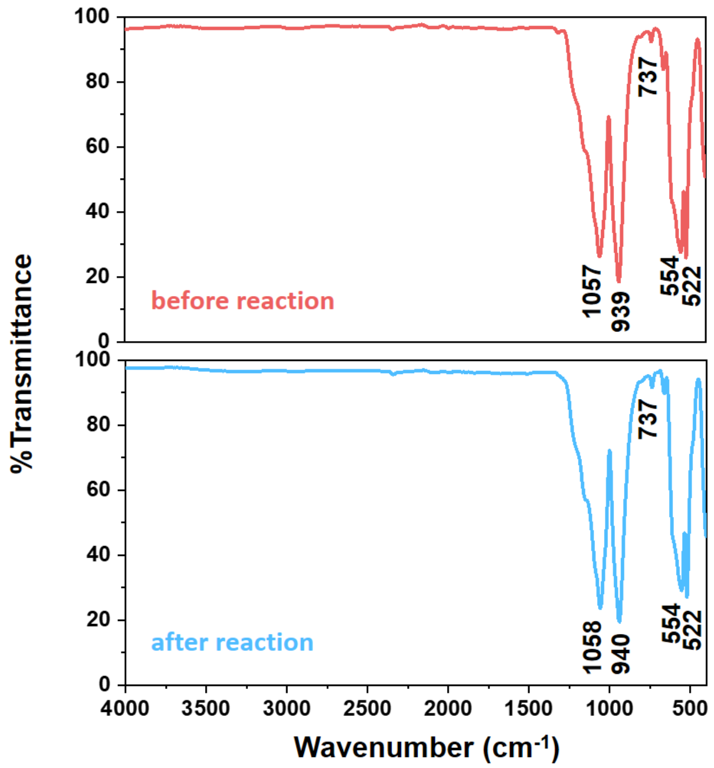


**Supplementary Fig. 19** FT-IR spectra of $Cu_2P_2O_7$ before and after 1 hour's $CO_2$RR at the applied potential of -1.40 V *vs*. RHE.

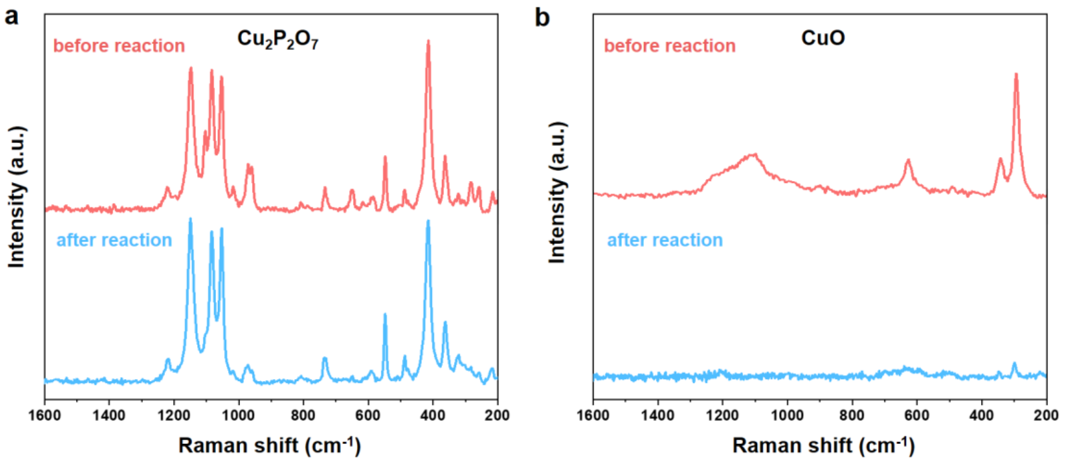


**Supplementary Fig. 20 a**, **b** Raman spectra of **a** $Cu_2P_2O_7$ and **b** CuO control sample before and after 1 hour's $CO_2$RR at the applied potential of -1.40 V *vs*. RHE.

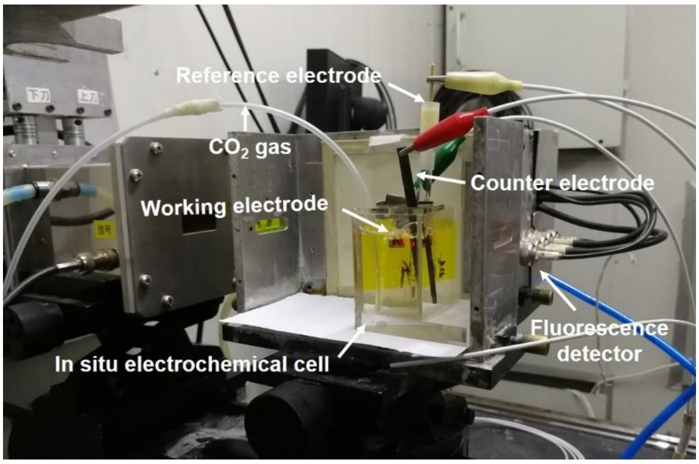


**Supplementary Fig. 21** Digital photograph of the in situ XAS electrochemical $CO_2$RR measurement system, in which the fluorescence model is adopted for collecting spectra.

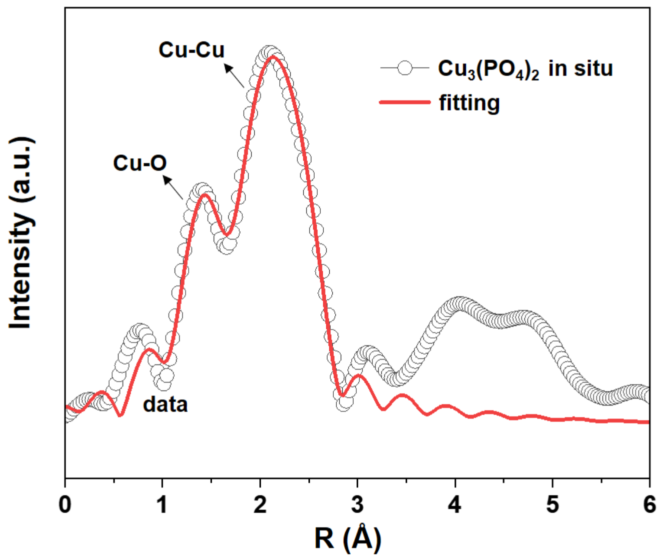


**Supplementary Fig. 22** Fourier-transformed Cu *K*-edge EXAFS spectrum of $Cu_3(PO_4)_2$ during $CO_2$RR and it's fitting result.

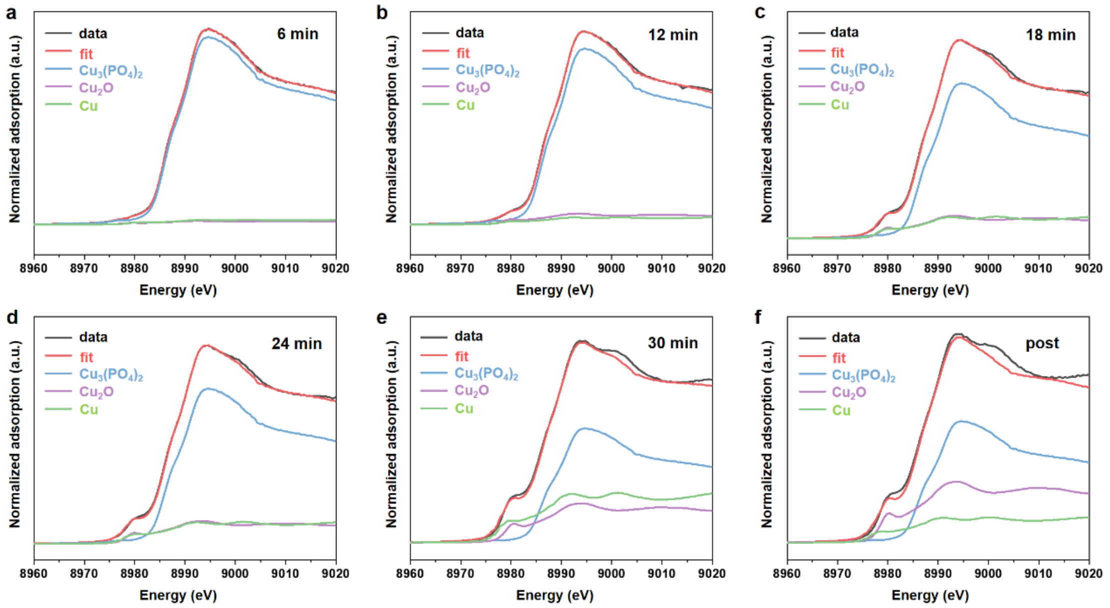


**Supplementary Fig. 23** LCF of XANES at Cu *K*-edge with respect to time during **a** 6 min, **b** 12 min, **c** 18 min, **d** 24 min, **e** 30 min of $CO_2$RR at - 1.45 V and **f** after reaction. CuPO, $Cu_2O$ and Cu foil are used as references to represent $Cu^{2+}$, $Cu^{1+}$ and $Cu^0$ species, respectively. The fitting results show that there are still 49% composed of $Cu^{2+}$ in $Cu_3(PO_4)_2$ after electrochemical reduction via $CO_2$RR for 30 min.

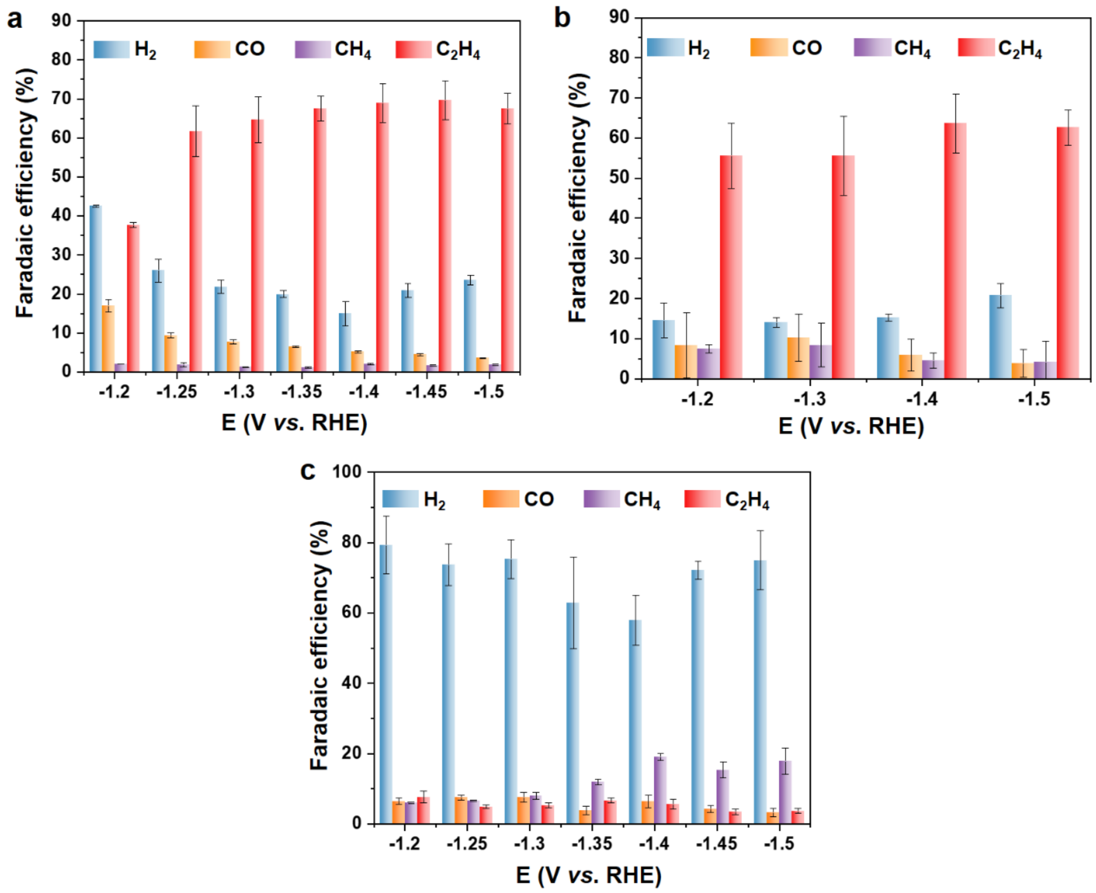


**Supplementary Fig. 24** Faradaic efficiencies of gas products of **a** $Cu_3(PO_4)_2$/GC, **b** $Cu_2P_2O_7$/GC and **c** CuO/GC at different applied potentials in $CO_2$-saturated 0.1 M $KHCO_3$, suggesting the superior $C_2H_4$ selectivity for CuPO. Error bars above were all based on the standard deviation of three measurements at each potential.

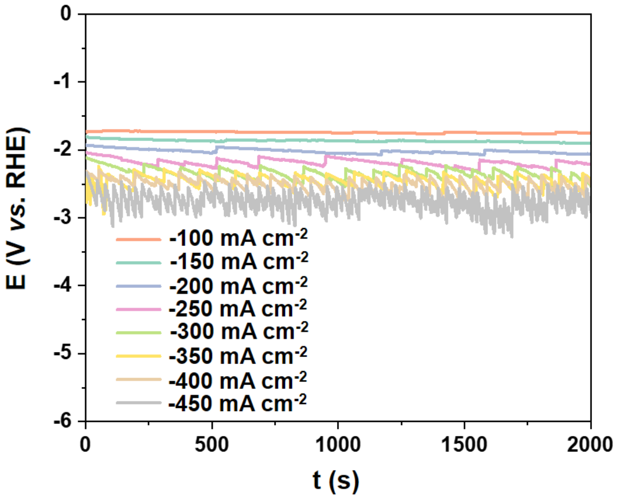


**Supplementary Fig. 25** Potential dependence on time during $CO_2$RR at -100~-450 mA cm$^{-2}$ in 2.0 M KOH aqueous electrolyte on the $Cu_3(PO_4)_2$/Cu/PTFE.

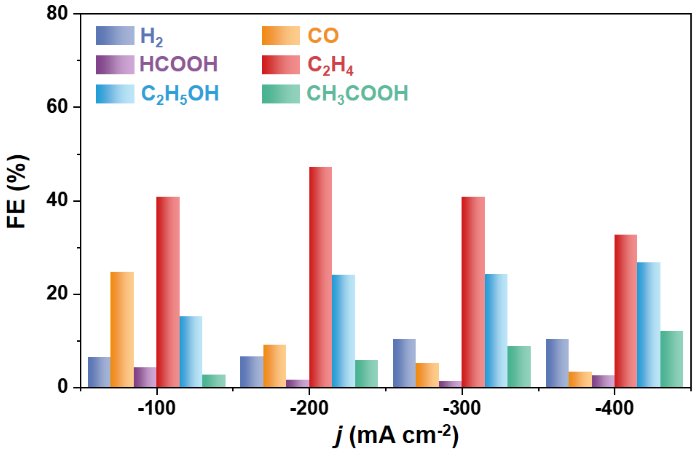


**Supplementary Fig.26** FEs for products over Cu/PTFE at various applied current densities in a flow cell reactor with 2.0 M KOH as the electrolyte.

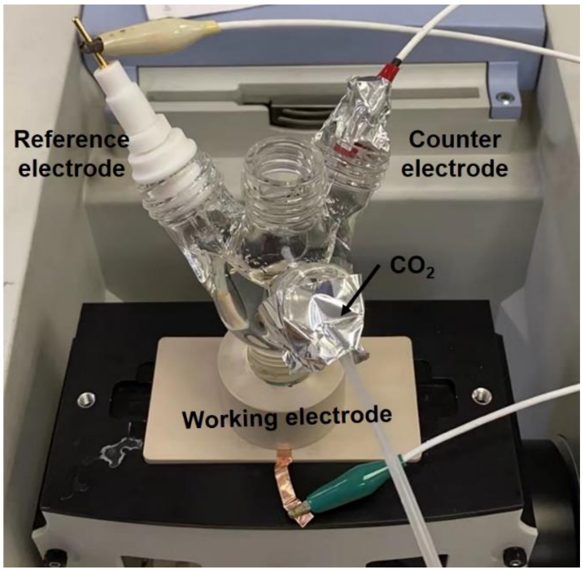


**Supplementary Fig. 27** Digital photograph of the homemade cell attached to the PerkinElmer spectrum 100 spectrometer for in situ SEIRAS measurement. The $CO_2$ gas flow rate through the gas chamber was controlled as 10 sccm by a gas flow controller.

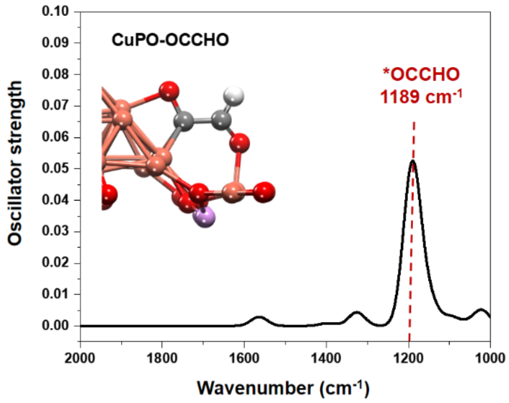


**Supplementary Fig. 28** Simulated Infrared spectrum of *OCCHO adsorbed on $Cu^0/Cu^{2+}$ ($Cu_3(PO_4)_2$) simulated by DFT frequency calculation. We conducted the frequency calculation in three directions and calculated Born charge for the related structure with the help of *Phonopy* package[1]. The post-processing was conducted and the diagram of IR was obtained through *IR-master* software.

**Supplementary Table 1** Cu *K*-edge EXAFS curves fitting parameters.

| Sample | Path | *N* | *R*(Å) | $\sigma^2$(10$^{-3}$ Å$^2$) | $\Delta E_0$(eV) | *R*-factor |
|---|---|---|---|---|---|---|
| $Cu_3(PO_4)_2$-in situ | Cu-O | 2.6 | 1.96 | 6.8 | -0.3 | 0.010 |
| | Cu-Cu | 4.6 | 2.56 | -6.2 | -2.8 | |

**Supplementary Table 2** $Cu^0$, $Cu^{1+}$, and $Cu^{2+}$ ratios and for the fitting of the linear combination of XANES spectra.

| **$Cu_3(PO_4)_2$ Cu oxidation state** | **Time (-1.45 V *vs*. RHE)** | | | | | |
|---|---|---|---|---|---|---|
| | **6 min** | **12 min** | **18 min** | **24 min** | **30 min** | **post** |
| $Cu^0$ | 0.04 | 0.06 | 0.16 | 0.23 | 0.28 | 0.08 |
| $Cu^{1+}$ | 0.01 | 0.06 | 0.12 | 0.14 | 0.24 | 0.41 |
| $Cu^{2+}$ | 0.95 | 0.88 | 0.72 | 0.63 | 0.49 | 0.51 |
| **CuO Cu oxidation state** | **Time (-1.45 V *vs*. RHE)** | | | | | |
| | **6 min** | **12 min** | **18 min** | **24 min** | **30 min** | **post** |
| $Cu^0$ | 0.04 | 0.18 | 0.62 | 0.68 | 0.73 | 0.69 |
| $Cu^{1+}$ | 0.00 | 0.20 | 0.13 | 0.13 | 0.13 | 0.17 |
| $Cu^{2+}$ | 0.96 | 0.63 | 0.25 | 0.19 | 0.13 | 0.13 |

**Supplementary Table 3** Faradaic efficiencies of gas products for CuPO/GC in 0.1 M $KHCO_3$.

| $Cu_3(PO_4)_2$/GC Faradaic efficiencies (%) | | | | | | |
|---|---|---|---|---|---|---|
| **Potential (V *vs.* RHE)** | **Current density (mA $cm^2$)** | **$H_2$** | **CO** | **$CH_4$** | **$C_2H_4$** | **Total all** |
| -1.20 | -18.1 | 42.9 | 18.7 | 2.0 | 37.9 | 101.6 |
| | -18.6 | 42.4 | 16.9 | 2.0 | 37.0 | 98.2 |
| | -18.6 | 42.4 | 15.4 | 2.1 | 38.2 | 98.2 |
| -1.25 | -19.1 | 29.3 | 8.7 | 2.4 | 54.2 | 95.1 |
| | -19.3 | 24.7 | 9.5 | 1.6 | 65.4 | 101.2 |
| | -19.6 | 24.0 | 10.0 | 1.5 | 65.6 | 101.1 |
| -1.30 | -23.7 | 22.9 | 8.1 | 1.2 | 58.0 | 90.1 |
| | -23.2 | 22.8 | 8.0 | 1.3 | 67.3 | 99.4 |
| | -23.5 | 20.0 | 7.1 | 1.3 | 68.4 | 96.7 |
| -1.35 | -27.3 | 20.9 | 6.7 | 1.3 | 63.8 | 92.7 |
| | -27.1 | 19.1 | 6.5 | 1.1 | 68.9 | 95.6 |
| | -27.1 | 19.8 | 6.4 | 1.1 | 69.7 | 97.0 |
| -1.40 | -29.7 | 18.6 | 5.4 | 2.2 | 63.2 | 89.5 |
| | -29.6 | 13.3 | 5.3 | 2.0 | 72.4 | 92.7 |
| | -29.7 | 13.0 | 4.9 | 2.0 | 71.3 | 91.5 |
| -1.45 | -33.0 | 20.1 | 4.6 | 1.8 | 63.9 | 90.4 |
| | -33.0 | 19.8 | 4.6 | 1.8 | 71.7 | 97.9 |
| | -33.1 | 23.0 | 4.2 | 1.4 | 73.4 | 102.0 |
| -1.50 | -36.7 | 24.4 | 3.7 | 1.9 | 63.0 | 93.1 |
| | -36.6 | 22.1 | 3.6 | 2.1 | 69.6 | 97.3 |
| | -36.7 | 24.0 | 3.4 | 1.6 | 70.0 | 99.0 |

| $Cu_2P_2O_7$/GC Faradaic efficiencies (%) | | | | | | |
|---|---|---|---|---|---|---|
| **Potential (V *vs.* RHE)** | **Current density (mA $cm^2$)** | **$H_2$** | **CO** | **$CH_4$** | **$C_2H_4$** | **Total all** |
| -1.20 | -14.7 | 19.5 | 17.8 | 6.3 | 46.8 | 90.4 |
| | -15.1 | 12.4 | 3.7 | 8.2 | 57.0 | 81.7 |
| | -15.4 | 11.6 | 3.7 | 8.0 | 62.9 | 86.8 |
| -1.30 | -20.1 | 14.5 | 13.1 | 5.7 | 48.8 | 82.6 |
| | -20.3 | 15.0 | 14.0 | 14.6 | 51.0 | 94.7 |
| | -20.7 | 12.7 | 3.4 | 4.7 | 66.8 | 87.6 |
| -1.40 | -26.3 | 15.8 | 10.4 | 6.7 | 55.3 | 88.4 |
| | -27.9 | 14.2 | 3.1 | 3.3 | 66.9 | 87.5 |
| | -28.0 | 15.7 | 4.0 | 3.6 | 69.7 | 92.9 |
| -1.50 | -33.0 | 17.4 | 7.6 | 10.1 | 57.9 | 92.9 |
| | -33.9 | 21.5 | 2.9 | 1.2 | 63.4 | 89.0 |
| | -34.3 | 23.3 | 1.0 | 1.2 | 66.5 | 92.1 |

**Supplementary Table 4** Faradaic efficiencies of gas products for CuO/GC in 0.1 M $KHCO_3$.

| **Potential (V *vs.* RHE)** | **Current density (mA cm$^2$)** | **$H_2$** | **CO** | **$CH_4$** | **$C_2H_4$** | **Total all** |
|---|---|---|---|---|---|---|
| -1.20 | -5.0 | 75.1 | 5.4 | 6.2 | 9.3 | 92.5 |
| | -5.1 | 74.0 | 6.8 | 6.0 | 7.7 | 94.6 |
| | -5.4 | 88.7 | 7.2 | 5.7 | 6.0 | 107.6 |
| -1.25 | -6.9 | 78.8 | 6.7 | 6.7 | 5.3 | 97.5 |
| | -6.9 | 67.2 | 7.8 | 6.7 | 4.3 | 85.9 |
| | -7.2 | 75.2 | 8.0 | 6.4 | 5.1 | 94.7 |
| -1.30 | -8.6 | 74.8 | 6.3 | 9.1 | 6.0 | 96.2 |
| | -8.4 | 70.1 | 7.4 | 7.4 | 5.0 | 89.8 |
| | -8.3 | 81.1 | 8.9 | 7.5 | 4.8 | 102.3 |
| -1.35 | -10.0 | 50.1 | 2.7 | 12.4 | 7.5 | 72.7 |
| | -10.1 | 62.4 | 3.8 | 12.3 | 6.4 | 85.0 |
| | -10.0 | 76.2 | 5.1 | 10.9 | 6.1 | 98.1 |
| -1.40 | -10.1 | 65.9 | 4.6 | 19.9 | 7.1 | 97.5 |
| | -9.7 | 55.2 | 6.4 | 19.2 | 5.3 | 86.1 |
| | -9.6 | 52.4 | 8.1 | 17.9 | 4.4 | 82.7 |
| -1.45 | -14.0 | 69.9 | 3.1 | 17.8 | 4.0 | 94.7 |
| | -13.9 | 74.9 | 4.5 | 13.5 | 3.7 | 96.5 |
| | -13.7 | 71.7 | 5.1 | 14.7 | 2.5 | 94.1 |
| -1.50 | -17.6 | 71.4 | 2.2 | 22.1 | 4.5 | 100.3 |
| | -17.0 | 69.0 | 3.0 | 16.5 | 3.3 | 91.7 |
| | -16.7 | 84.6 | 4.4 | 14.9 | 3.2 | 107.1 |

**Supplementary Table 5** Maximum $FE_{C2+}$, $j_{C2+}$ and the corresponding potential of CuPO, and some recently reported outstanding $CO_2$-to-$C_{2+}$ electrocatalysts.

| Catalyst | Electrolyte | $FE_{C2+}$ (%) | $j_{C2+}$ (mA cm$^2$) | Reference |
|---|---|---|---|---|
| **CuPO/Cu/PTFE** | **2.0 M KOH** | **90.9** | **-318.2** | **This work** |
| Cu-Pd[2] | 0.5 M $K_2SO_4$ (pH=2) | 89 | -445 | *Nat. Catal.* **5**, 564−570 (2022) |
| $Cu(OH)_2$/Cu foil[3] | 1 M KOH | 87 | -217 | *Angew. Chem. Int. Ed.* **60**, 4879−4885 (2021) |
| F-Cu[4] | 0.75 M KOH | 85.8 | -1372.8 | *Nat. Catal.* **3**, 478−487 (2020) |
| reconstructed Cu[5] | 3 M KOH | ~84 | -336 | *Adv. Mater.* **30**, 1804867 (2018) |
| Cu/GDL[6] | 3 M KCl | 82.7 | -97.3 | *J. Am. Chem. Soc.* **143**, 3245−3255 (2021) |
| | 2 M KCl | 80.4 | -120.6 | |
| $dCu_2O/Ag_{2.3\%}$[7] | 1 M KOH | 82.1 | -656.8 | *Nat. Commun.* **13**, 3754 (2022) |
| CuPb-0.7/C[8] | 1 M KOH | 81.6 | -326.4 | *ACS Nano* **15**, 1039–1047 (2021) |
| Cu_I[9] | 0.1 M $KHCO_3$ | ~80 | ~-31.2 | *Angew. Chem. Int. Ed.* **58**, 17047−17053 (2019) |
| B-Cu-Zn[10] | 1 M KOH | 79 | -158 | *Angew. Chem. Int. Ed.* **60**, 9135−9141 (2021) |
| Cu(B)-2[11] | 0.1 M $KHCO_3$ | 79 | -55 | *Nat. Chem.* **10**, 974−980 (2018) |
| Cu-FEP[12] | 1 M KOH | ~77 | ~-600 | *Adv. Energy Mater.* **12**, 2103663 (2022) |
| monodispersed $Cu_2O$ NPs[13] | 1 M $KHCO_3$ | 75.5 | -226.5 | *Joule* **4**, 1−17 (2020) |
| | | 68.4 | -342 | |
| multi-hollow $Cu_2O$[14] | 2 M KOH | 75.2 | -267 | *J. Am. Chem. Soc.* **142**, 6400–6408 (2020) |
| OD-Cu[15] | 1 M $KHCO_3$ | 74.9 | -224.7 | *J. Am. Chem. Soc.* **144**, 259−269 (2022) |
| N-Cu[16] | 1 M KOH | 73.7 | -810.7 | *J. Am. Chem. Soc.* **144**, 14936–14944 (2022) |
| $Cu_2P_2O_7$[17] | 0.1 M KOH | 73.6 | -257.6 | *Angew. Chem. Int. Ed.* **61**, e20211438 (2022) |
| Cu-CuI[18] | 1 M KOH | 71 | -591 | *Angew. Chem. Int. Ed.* **60**, 14329−14333 (2021) |
| Cu-D[19] | 1 M KOH | 64 | -255 | *J. Am. Chem. Soc.* **143**, 8011−8021 (2021) |

**Supplementary Note 1.** Data extraction.

In the preparation phase of extracting materials from the Materials Project (MP) database, we obtained all materials containing $Cu^{2+}$ used the pymatgen package with Python code[20]. Here, in the potential candidates, one metal element Cu and the non-metal elements (B, C, N, O, F, Si, P, S, Cl, Br, I, Se) are considered. A total of 83 candidates were obtained for the further high-throughput screening.

**Supplementary Note 2.** Calculation of electrochemical stability.

Here we calculated the dissolution potential ($U_{diss}$) to examine the electrochemical stability of candidate materials, in which all candidates were considered to be reduced to Cu metal and corresponding acid ions. The pourbaix diagrams in MP were employed to determine the energy of acid ion that could stably exist for each nonmetallic element in a mild acid environment at an applied potential of about 1.0 V. Taking $Cu_2P_2O_7$ as an example, corresponding to the acid ion species $H_2PO_4^-$, the dissolution reaction equation is shown as follows:

$$1/2Cu_2P_2O_7 + 1/2H_2O + H^+ + 2e^- \rightarrow Cu + H_2PO_4^- \quad (1)$$

The effect of the potential on the state with an electron involved was considered by shifting the energy of -e$U$. Thus, the Gibbs free reaction energy from $Cu_2P_2O_7$ to Cu and $H_2PO_4^-$can be written as follows:

$$\Delta G = E(Cu) + G(H_2PO_4^- - e^-) - 1/2E(Cu_2P_2O_7) - 1/2G(H_2O) - G(H^+ + e^-) + 2eU \quad (2)$$

where $E(Cu_2P_2O_7)$ and $E(Cu)$ are total energies from the DFT calculations. $G(H_2PO_4^- - e^-)$ was calculated as below:

$$G(H_2PO_4^- - e^-) = 1.29 + G(H_3PO_4) - G(H^+ + e^-) \quad (3)$$

Noticeably, 1.29 eV is the standard Gibbs free energy for $H_3PO_4$ hydrolysis ($H_3PO_4 \rightarrow H^+ + H_2PO_4^-$). The standard Gibbs free energy of $H_3PO_4 \rightarrow H^+ + H_2PO_4^-$ at 298 K can be defined as $\Delta G° = -RT\text{In}K°$, where $K°$ is the equilibrium constant of $H_3PO_4 \rightarrow H^+ + H_2PO_4^-$ ($K°$ = 0.0071, $T$ = 298 K). $G(H^+ + e^-)$ and the chemical potential of half of a $H_2$ molecule are equilibrated at 0 V at all pH values in the computational hydrogen electrode (CHE) model[21]. The Gibbs free energy of $H_3PO_4$ ($G(H_3PO_4)$) is equal to the formula:

$$G(H_3PO_4) = E(H_3PO_4) - TS \quad (4)$$

where $E(H_3PO_4)$ is the total energy of $H_3PO_4$ from MP, $TS$ is the entropy at room temperature $T$ = 298 K.

Therefore, we can calculate the dissolution potential while $\Delta G$ = 0, and the corresponding formula is:

$$U_{diss} = -(E(Cu) + G(H_2PO_4^- - e^-) - 1/2E(Cu_2P_2O_7) - 1/2G(H_2O) - G(H^+ + e^-)) / 2e \quad (5)$$

**Supplementary Note 3.** Materials.

Copper nitrate trihydrate ($Cu(NO_3)_2·3H_2O$) was purchased from Aladdin, and Ammonium dihydrogen phosphate ($NH_4H_2PO_4$) was obtained from Sinopharm Chemical Reagent Co., Ltd. Citric acid monohydrate ($C_6H_{10}O_8$) was purchased from Shanghai Lingfeng Chemical Reagent Co., Ltd. Ammonium fluoride ($NH_4F$), carbon paper (TGP-H-060) and Nafion 117 proton exchange membrane were bought from Alfa Aesar. Potassium hydroxide (KOH), Potassium bicarbonate ($KHCO_3$) and iso-propyl alcohol (AR, ≥99.0%) were obtained from Shanghai Chemical Reagent Co., Ltd. Deuterium oxide ($D_2O$) was bought from J&K Scientific Ltd. Dimethyl sulfoxide (DMSO, $C_2H_6OS$), Nafion (5 wt% in a mixture of lower aliphatic alcohols and water) was obtained from Sigma-Aldrich. Carbon dioxide ($CO_2$, 99.9999%) was bought from Shanghai Jiajie Special Gas Co., Ltd. All reagents were commercially available as analytical grade. All water used was purified with a Millipore system (typically 18.2 MΩ cm resistivity).

**Supplementary References**


1. Togo, A. & Tanaka, I. First principles phonon calculations in materials science. *Scripta Mater.* **108**, 1−5 (2015).
2. Xie, Y. et al. High carbon utilization in $CO_2$ reduction to multi-carbon products in acidic media. *Nat. Catal.* **5**, 564−570 (2022).
3. Zhong, D. et al. Coupling of Cu(100) and (110) facets promotes carbon dioxide conversion to hydrocarbons and alcohols. *Angew. Chem. Int. Ed.* **60**, 4879−4885 (2021).
4. Ma, W. et al. Electrocatalytic reduction of $CO_2$ to ethylene and ethanol through hydrogen-assisted C–C coupling over fluorine-modified copper. *Nat. Catal.* **3**, 478−487 (2020).
5. Kibria, M. G. et al. A surface reconstruction route to high productivity and selectivity in $CO_2$ electroreduction toward $C_{2+}$ hydrocarbons. *Adv. Mater.* **30**, 1804867 (2018).
6. Zhang, X. et al. Selective and high current $CO_2$ electro-reduction to multicarbon products in near-neutral KCl electrolytes. *J. Am. Chem. Soc.* **143**, 3245−3255 (2018).
7. Wang, P. et al. Boosting electrocatalytic $CO_2$–to–ethanol production via asymmetric C–C coupling. *Nat. Commun.* **13**, 3754 (2022).
8. Wang, P. et al. Synergized Cu/Pb core/shell electrocatalyst for high-efficiency $CO_2$ reduction to $C_{2+}$ liquids. *ACS Nano* **15**, 1039−1047 (2020).
9. Gao, D. et al. Selective $CO_2$ electroreduction to ethylene and multicarbon alcohols via electrolyte-driven nanostructuring. *Angew. Chem. Int. Ed.* **58**, 17047−17053 (2019).
10. Song, Y. et al. B-Cu-Zn Gas Diffusion Electrodes for $CO_2$ Electroreduction to $C_{2+}$ products at high current densities. *Angew. Chem. Int. Ed.* **60**, 9135−9141 (2021).
11. Zhou, Y. et al. Dopant-induced electron localization drives $CO_2$ reduction to $C_2$ hydrocarbons. *Nat. Chem.* **10**, 974−980 (2018).
12. Pham, T. H. M. et al. Enhanced electrocatalytic $CO_2$ reduction to $C_{2+}$ products by adjusting the local reaction environment with polymer binders. *Adv. Energy Mater.*

**12**, 2103663 (2022).

13. Miao, R. K. et al. Electroosmotic flow steers neutral products and enables concentrated ethanol electroproduction from $CO_2$. *Joule* **5**, 2742−2753 (2021).
14. Wu, Z.-Z. et al. Identification of Cu(100)/Cu(111) interfaces as superior active sites for CO dimerization during $CO_2$ electroreduction. *J. Am. Chem. Soc.* **144**, 259−269 (2022).
15. Chen, Z. et al. Grain-boundary-rich copper for efficient solar-driven electrochemical $CO_2$ reduction to ethylene and ethanol. *J. Am. Chem. Soc.* **142**, 6878−6883 (2020).
16. Zheng, M. et al. Electrocatalytic $CO_2$-to-$C_{2+}$ with ampere-level current on heteroatom-engineered copper via tuning *CO intermediate coverage. *J. Am. Chem. Soc.* 144, 14936−14944 (2022).
17. Sang, J. et al. A reconstructed $Cu_2P_2O_7$ catalyst for selective $CO_2$ electroreduction to multicarbon products. *Angew. Chem. Int. Ed.* **61**, e202114238 (2022).
18. Li, H. et al. High-rate $CO_2$ electroreduction to $C_{2+}$ products over a copper-copper iodide catalyst. *Angew. Chem. Int. Ed.* **60**, 14329−14333 (2021).
19. Niu, Z.-Z. et al. Hierarchical copper with inherent hydrophobicity mitigates electrode flooding for high-rate $CO_2$ electroreduction to multicarbon products. *J. Am. Chem. Soc.* **143**, 8011−8021 (2021).
20. Jain, A. et al. Commentary: the materials project: a materials genome approach to accelerating materials innovation. *APL Mater.* **1**, 011002 (2013).
21. Nørskov, J. K. et al. Origin of the overpotential for oxygen reduction at a fuel-cell cathode. *J. Phys. Chem. B* **108**, 17886 (2004).
